# Quantifying Fenton reaction pathways driven by self-generated H_2_O_2_ on pyrite surfaces

**DOI:** 10.1038/srep43703

**Published:** 2017-03-06

**Authors:** C. Gil-Lozano, A. F. Davila, E. Losa-Adams, A. G. Fairén, L. Gago-Duport

**Affiliations:** 1 Centro de Astrobiología (CSIC-INTA), 28850 Torrejón de Ardoz, Madrid, Spain; 2 Carl Sagan Center at the SETI Institute, 189 Bernardo Avenue, Suite 100, Mountain View, CA 94043, USA; 3 Departamento de Geociencias Marinas, Universidad de Vigo, Lagoas Marcosende, 36310-Vigo, Spain; 4 Department of Astronomy, Cornell University, Ithaca, 14853 NY, USA

## Abstract

Oxidation of pyrite (FeS_2_) plays a significant role in the redox cycling of iron and sulfur on Earth and is the primary cause of acid mine drainage (AMD). It has been established that this process involves multi-step electron-transfer reactions between surface defects and adsorbed O_2_ and H_2_O, releasing sulfoxy species (e.g., S_2_O_3_^2−^, SO_4_^2−^) and ferrous iron (Fe^2+^) to the solution and also producing intermediate by-products, such as hydrogen peroxide (H_2_O_2_) and other reactive oxygen species (ROS), however, our understanding of the kinetics of these transient species is still limited. We investigated the kinetics of H_2_O_2_ formation in aqueous suspensions of FeS_2_ microparticles by monitoring, in real time, the H_2_O_2_ and dissolved O_2_ concentration under oxic and anoxic conditions using amperometric microsensors. Additional spectroscopic and structural analyses were done to track the dependencies between the process of FeS_2_ dissolution and the degradation of H_2_O_2_ through the Fenton reaction. Based on our experimental results, we built a kinetic model which explains the observed trend of H_2_O_2_, showing that FeS_2_ dissolution can act as a natural Fenton reagent, influencing the oxidation of third-party species during the long term evolution of geochemical systems, even in oxygen-limited environments.

The chemical and electrical properties of pyrite (FeS_2_) – the most common iron disulfide in the Earth’s crust – make it a promising material for the construction of photovoltaic panels^1^, as well as for wastewater remediation^2^,^3^,^4^,^5^,^6^,^7^. In the last decades, a number of experimental and field studies have addressed the oxidative dissolution of pyrite, providing an accurate characterization of the process^8^,^9^,^10^,^11^,^12^,^13^,^14^,^15^,^16^,^17^. Recent investigations have shown that, aside from the iron and sulfoxy species released during pyrite dissolution, ROS are always present as transient by-products, both under oxic and anoxic conditions^18^,^19^,^20^,^21^,^22^,^23^,^24^,^25^,^26^, and providing an important pathway to oxidize third-party species through oxygen evolution reaction (OER)^18^,^27^,^28^,^29^,^30^.

The rupture of S-Fe and S-S bonds over pyrite surface by mechanical fracture or during the dissolution process induces the formation of the non-stoichiometric defect sites (*i.e.,* the dangling bonds) that trigger adsorption reactions^31^,^32^,^33^. The iron surface species formed cause a reduction in the band gap of the pyrite from 0.86 electron volts (eV) in the bulk to 0.55 eV on the surface^34^, catalyzing the dissociation of adsorbed oxygen (O_2_) and water (H_2_O) molecules and leading to the formation of H_2_O_2_ (equations 1,2,3,4)^19^,^25^,^26^,^35^:


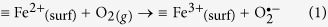



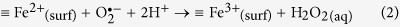



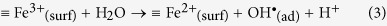



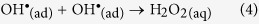


At the same time, pyrite releases Fe^2+^into solution than can catalyze the Fenton and Haber-Weiss reactions, leading to the generation of other ROS (equations 5)^4^,^22^,^25^,^26^:


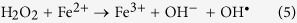



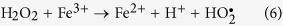



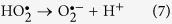



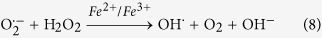


Although this sequence of reactions describes the basic features of H_2_O_2_ formation during pyrite dissolution (equations 1,2,3,4) and its further degradation to secondary ROS in solution (equations 5,6,7,8), various important aspects remain unclear, for instance: (1) can free radicals be formed by mechanism other than the photoactivation (i.e. by mechanical bond fracture or non-stoichiometry dissolution of pyrite)? (2) can the formation of H_2_O_2_ occur in strictly anoxic conditions overcoming the energy required to split the water molecule and the further release of O_2_? and (3) can the process of ROS generation by pyrite be sustained over long periods of time?.

Real-time measurements of this process are made difficult by ROS reactivity and the subsequent redox transformations of iron and sulfur species. Spectroscopic and fluorescence methods are commonly used to measure the concentration of H_2_O_2_; however, these methods usually need the presence of dyes or chelating agents that are not well suited for the kinetic analysis of transient phases. In this study, we measured the real-time generation and decomposition of H_2_O_2_ and dissolved O_2_ induced by pyrite surfaces under different boundary conditions (i.e., dark/light, oxic/anoxic) to investigate the kinetic role of ROS during pyrite dissolution. In addition, we analyzed both the chemical evolution of dissolved species and surface pyrite oxidation with spectroscopy (UV-Vis, XPS), Cyclic Voltammetry (CV) and high-resolution transmission electron microscopy (HTREM) and performed specific experiments to evaluate the persistence of H_2_O_2_ formation. Based on our results, we developed a kinetic model for the coupling between pyrite dissolution and H_2_O_2_ generation/degradation through the Fenton reaction. When combined with the observed trends this model leads to the definition of constraints on the overall process of ROS oxidation mechanism induced by pyrite surfaces.

## Results

### H_2_O_2_ and O_2_ evolution: general pathway

**Figure 1:**
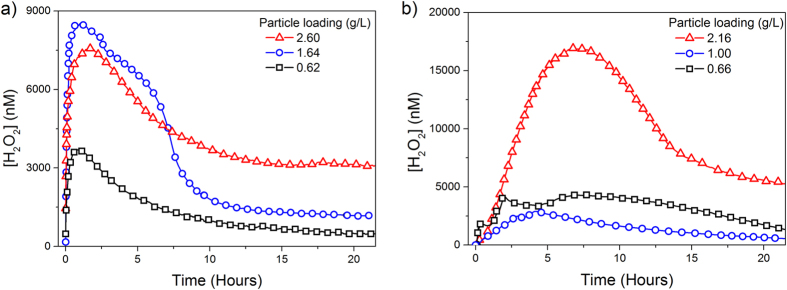
H_2_O_2_ evolution induced by pyrite slurries in unbuffered water. Inset: particle loading (g/L) (**a)** oxic conditions, (**b)** anoxic conditions.

Figure 1 shows the concentration of H_2_O_2_ as a function of time in aqueous suspensions of pyrite starting at circumneutral pH, under oxic and anoxic conditions. H_2_O_2_ concentration increased at the beginning of the experiment until a maximum value was reached, and decreased thereafter asymptotically towards a nearly stationary, residual value ([H_2_O_2_]>200 nM) still measurable at the end of the experiment (~22 h). This coupled generation-decay response was generally observed in every experiment, although there were variations in the particular shape of the curves and, some experiments showed characteristic shoulders or secondary maxima at intermediate stages of the process. The overall process followed a sigmoidal trend, suggesting a strong interaction between the H_2_O_2_ generation and degradation rates characteristic of an autocatalytic process. H_2_O_2_ formation and decomposition evolved more slowly under anoxic than under oxic conditions. We did not observe any correlation between pyrite loading and the H_2_O_2_ yield between experimental runs under our experimental conditions (i.e., 0.5–0.3 g/L, unbuffered neutral pH) because of several factors (e.g., kinks, steps, lattice anisotropy) can determine the variability of the reactive surface between samples^36^,^37^,^38^,^39^.

**Figure 2:**
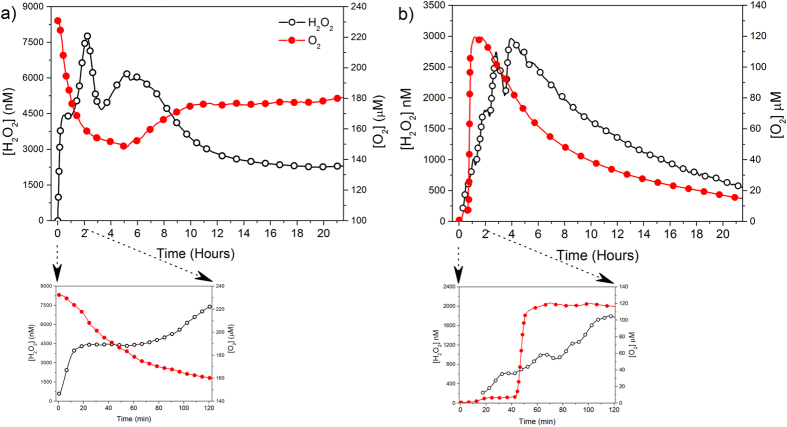
Simultaneous trends of O_2_ and H_2_O_2_ obtained from pyrite slurries in unbuffered water. The bottom figure shows the first two hours of the reaction, under (**a)** oxic (particle loading = 1.75 g/l) and (**b)** anoxic conditions (particle loading = 1.09 g/L).

The evolution of O_2_ under oxic conditions was characterized by an asymptotic decrease followed by a slight increase and a steady stable period at the end of the experiment (Supplementary Fig. S1). The opposite trend was observed under anoxic conditions (i.e., an initial increase in O_2_ followed by an asymptotic decrease). To clarify the role of O_2_ over the formation of H_2_O_2_, we monitored simultaneously the evolution of O_2_ and H_2_O_2_ under oxic and anoxic conditions (Fig. 2). In oxic conditions (Fig. 2a), the amount of H_2_O_2_ increased whereas O_2_ was rapidly consumed at the beginning of the experiment. Under anoxic conditions O_2_ and H_2_O_2_ formed concomitantly (Fig. 2b).

During the experiments, pH became acidic under both oxic and anoxic conditions. Overall, pH dropped rapidly towards a nearly constant value approximately 2 to 3 pH units lower then at the initial pH value. The initial drop was more pronounced under anoxic than under oxic conditions (2 hours vs 10 hours, respectively), and in both cases the initial decline in pH values was accelerated with increasing pyrite loading (Supplementary Fig. S2).

### The Fenton reaction in anoxic conditions: spectroscopic experiments

**Figure 3:**
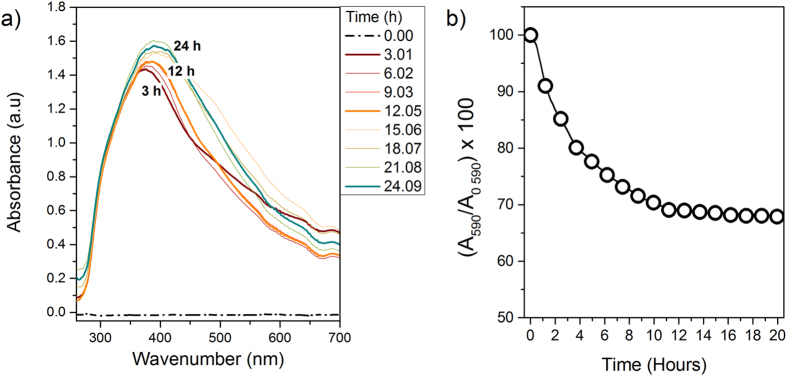
Spectroscopic monitoring of Fe^3+^and OH^**·**^ from pyrite slurries under anoxic conditions. (**a**) Absorption bands detected from aqueous pyrite suspension in anoxic conditions and in the dark showing Fe^3+^-complexes signatures (particle loading = 0.28 g/L). The numbers inserted over the absorption bands show the reaction time. Spectra were registered in real time using a liquid waveguide capillary flow cell (LWCC; path length: 250 cm; WPI), connected to the batch reactor by a peristaltic pump; (**b**) Degradation of CV solution upon pyrite aqueous reaction under anoxic conditions and in the dark (particle loading = 0.12 g/L, [CV]_0_ = 225.5 μM).

To determine if the Fenton reaction was actually the mechanism to degrade the H_2_O_2_ forming under anoxic conditions, we monitored the production of Fe^3+^and OH^**·**^ species in pyrite suspensions under anoxic conditions and in the dark (Fig. 3). First, we evaluated the intensity of the absorbance bands in the UV-Vis range of 260–700 nm to identify the formation of dissolved Fe^3+^-complexes that coexist in solution in the range 300–450 nm^40^,^41^,^42^,^43^ (Fig. 3a). The first absorbance peak (λmax ~375 nm) evolved after three hours of reaction and was shifted to higher wavelengths (around 390–400 nm), as the reaction proceeded. Second, we monitored the formation of short-lived radicals, mainly OH^**·**^ by measuring the decrease in the light absorption spectrum (λmax = 590 nm) of Crystal Violet used as a dye probe^44^ (Fig. 3b). The absorption spectrum of CV showed a rapid decline indicating that OH^**·**^ formation occurs concomitantly from the start-up of the experiment.

### Tracking the reversibility of H_2_O_2_ generation: cycling experiments

**Figure 4:**
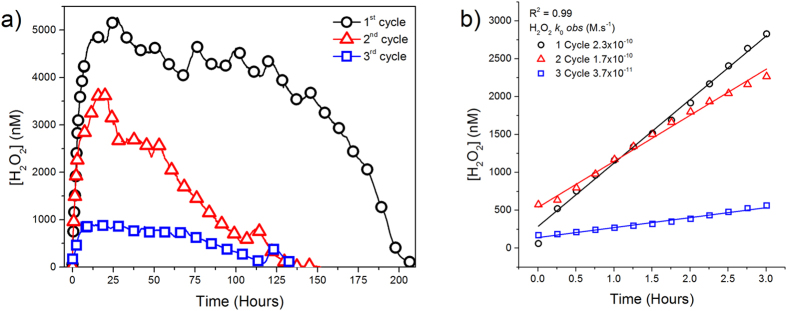
Recurrence of H_2_O_2_ formation by pyrite slurry after H_2_O renewal. (**a)** H_2_O_2_ evolution from the same pyrite slurry after renewing H_2_O twice, in oxic-open conditions (pyrite load particle = 0.33 g/L, ΔpH_1_cycle = 6.8–3.8, ΔpH_2_cycle = 6.8–6.1, ΔpH_3_cycle = 6.8–6.7); (**b)** Initial observed rate of H_2_O_2_ formation assuming a zero kinetic order rate.

We performed these experiments to better understand the long-term evolution of H_2_O_2_ during pyrite dissolution in an open system. In order to use the same physical pyrite particles and in the same geometry, pyrite microparticles were adhered over silicone strips to form a thin film which was placed on the internal wall of the reactor as in the spectroscopic experiments. We monitored the evolution of H_2_O_2_ until the observable amount of H_2_O_2_ attained a constant value or a concentration of zero (Fig. 4). At that point, we replaced the solution inside the batch reactor with distilled H_2_O, and monitored the evolution of H_2_O_2_ again (we repeated this procedure twice). After a full cycle of H_2_O_2_ generation and decay, the formation of H_2_O_2_ was found to resume after the addition of fresh distilled H_2_O, although the maximum H_2_O_2_ yield was consistently lower than in the previous cycle (Fig. 4a). Assuming a zero-order kinetic model, the initial observed rate of the H_2_O_2_ formation (*k*_*0 obs*_) showed a slight decrease between the first and the second cycle, which was more prevalent in the third cycle (Fig. 4b). This decrease could be attributed to the formation of oxide patches on pyrite surface that partially block some of the iron reactive centers.

### Surface characterization (CV, XPS and HRTEM)

**Figure 5:**
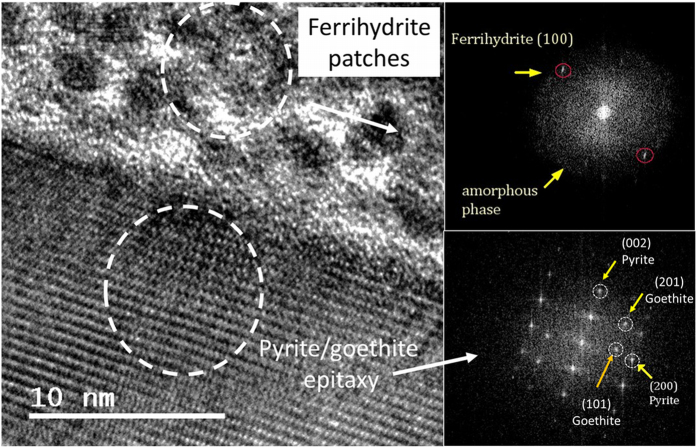
Identification of iron oxide patches in pyrite surface by HTREM. (**a)** HTREM image showing the formation of secondary products over a lamella of pyrite after 22 hours immersed in a micromolar solution of H_2_O_2_ under anoxic conditions; (**b)** FFT of ferrihydrite patches; (**c)** FFT of the crystalline part showing spacing characteristic of pyrite and goethite.

Results of cyclic voltammetry under anoxic conditions revealed the anodic peaks associated with H_2_O oxidation by one electron and by two electron transfer (Supplementary Fig. S3). Interestingly, in the cathodic counterpart, the peak assigned to iron reduction is split into two peaks (0.1 V NHE and 0.2VNHE, respectively), indicating that a fraction of ≡Fe^3+^is reduced in a nearly spontaneous manner. The analyzed XPS spectra of the (001) face of pyrite after aqueous reaction in the presence and absence of dissolved O_2_ (g) resulted in significant differences, showing major surface oxidation under oxic conditions with the subsequent formation of patches (Supplementary Fig. S4). In fact, XPS analysis of the (100) face of the pyrite after anoxic reaction only showed appreciable changes in the O1s orbital showing a shift to lower binding energies associated with an increase of the hydroxyl contribution (−OH) (Supplementary Fig. S5). In order to facilitate the identification of ≡S^2−^ and ≡Fe^3+^dangling bonds, one sample was ion-sputtered, which promoted the breakage of the S-S dimers (as in the grinding procedure) (Supplementary Fig. S6). The formation of iron oxidation patches was also observed with HRTEM. Figure 5 shows an image of the pyrite surface after 22 hours of reaction in a micromolar solution of H_2_O_2_ in absence of O_2_(g). The presence of discrete oxidation patches was observed in the uppermost area of the micrograph (Fig. 5a). The FFT (Fast Fourier transform) proved that the lattice fringe spacing of low contrast clusters (~0.25 nm) were consistent with ferrihydrite nanocrystals (Fig. 5b), viewed down [001]. Additional HRTEM images (Supplementary Fig. S7) also showed the interplanar spacing characteristic of two-line ferrihydrite^45^,^46^. The FFT of the crystalline part showed the interplanar spacing of pyrite but also of goethite, suggesting that ferrihydrite can be a precursor of goethite formation (Fig. 5c)^47^.

### Fitting the experimental data: kinetic modeling of H_2_O_2_ evolution

Our experimental data suggests that the observed trend of H_2_O_2_ is the result of the coupling between the H_2_O_2_ generation by iron defect sites on the pyrite surface and the H_2_O_2_ decomposition by the Fenton reaction. Based on these results, we built a kinetic model that allowed us to analyze the process in terms of elementary reactions and to determine the specific rate constant of the H_2_O_2_ formation in oxic and anoxic conditions by fitting the experimental data. In summary, the model describes the net amount of H_2_O_2_ according with the following expression (Supplementary, Modeling approaches):


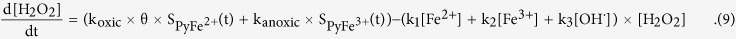


Where k_1_, k_2_ and k_3_, represent the rate constants of each Fenton reaction step (Supplementary, Table S1). As shown in the equation, the H_2_O_2_ formation was calculated assuming a first order dependence on the reactive surface and, both iron defect sites act simultaneously, but in anoxic conditions the H_2_O_2_ produced by Fe^2+^sites is limited by the O_2_ derived by the Fenton reaction. Minimization between experimental curves and the values calculated from equation 9 were made with a non-linear least squares approach using the Marquardt algorithm, with k_oxic_ and k_anoxic_, the specific rate constants of H_2_O_2_ surface generation, as adjustable parameters (Table 1).

**Figure 6:**
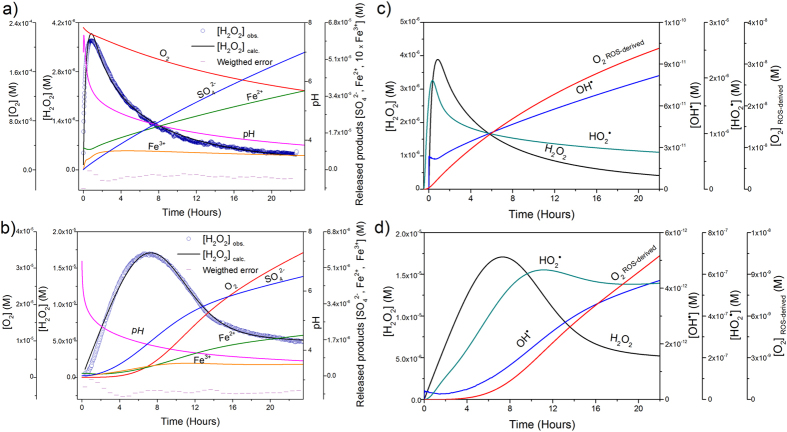
Experimental and fitting curves of H_2_O_2_ evolution, together with model-derived trends of pyrite dissolution products and secondary ROS. (**a**) experimental and model curves of H_2_O_2_ together with Fe^2+^/ Fe^3+^, SO_4_^2−^, pH and O_2_ model trends, under oxic conditions (pyrite particle loading = 0.71 g/L, A/V_0_ = 1 m^2^/L, pH_0_ = 7, [O_2_]_0_ = 232 μM); and, (**b**) anoxic conditions (pyrite particle loading = 2.16 g/L, A/V_0_ = 3.16 m^2^/L, pH_0_ = 7, [O_2_]_0_ = 0 μM). Time evolution of ROS obtained with the previous models under: (**c**) oxic conditions; and (**d**) anoxic conditions. ^*^The evolution of O_2_^**·**−^ was omitted from the plot because model calculations gave negligible concentrations.

Figure 6 presents the fitted values corresponding to the evolution of H_2_O_2_ in oxic and anoxic conditions together with the model derived results. Under oxic conditions (Fig. 6a), when pH was higher, the ferrous surface iron was oxidized by dissolved O_2_ forming H_2_O_2_ according the first order rate. Deviations of the linearity started to occur due to a change in the reaction stoichiometry by the simultaneous decrease in pH and increase in dissolved Fe^2+^and H_2_O_2_. From this point, the Fenton reaction became important and the oxidation rate of Fe^2+^by O_2_ slowed down leading to a minor O_2_ consumption. As a result, reactions forming ROS -catalyzed by Fe^2+^and Fe^3+^− became effective for H_2_O_2_ degradation, which rapidly decreased, while iron species followed an opposite trend. Under anoxic conditions (Fig. 6b), the formation of H_2_O_2_ proceeded more slowly. The decomposition of H_2_O_2_ was also retarded because the concentration of dissolved [Fe^2+^] supplied to solution by pyrite dissolution was lower. The Fenton reaction was initiated when pH values dropped below 4.5 and the ratio H_2_O_2_/Fe^2+^increased, catalyzing the H_2_O_2_ decomposition similar to the oxic experiments. The analysis of ROS derived from the model in both oxic and anoxic conditions (Fig. 6c and d) shows that, the first reactive species formed was OH^**·**^ acting as a chain initiator, forming additional free radicals. The majority of the OH^**·**^ reacted with H_2_O_2_, generating HO_2_^**·**^, the conjugate acid of O_2_^**·**−^, which is the limiting reagent to assist the redox cycling of Fe^3+^/Fe^2+^forming O_2_ and helping to buffer the pH drop

**Figure 7:**
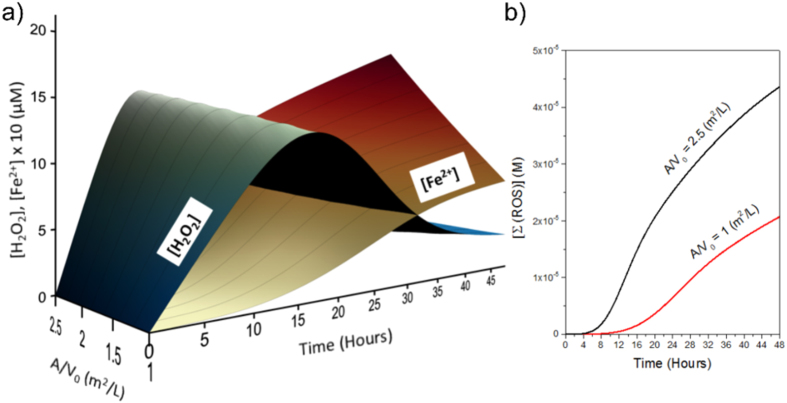
Influence of pyrite reactive surface area on H_2_O_2_ evolution. (**a)** 3D plot showing the H_2_O_2_ and Fe^2+^simulated trends at different values of the surface area of pyrite per volume of H_2_O (A_0_/V from 1 to 2.5, m^2^/L). (**b)** Summation of ROS generated by the Fenton reaction for the lowest and the highest values of the reactive surface (Initial conditions: *k*_oxic_ = 2.33 × 10^−4^, *k*_anoxic_ = 6.46 × 10^−9^ mol/m^2^s –average values of the rate constants estimated by the model-, pyrite particle loading = 1 g/L, pH_0_ = 7, [O_2_]_0_ = 0 μM).

Contrary to the expectation, the maximum amount of H_2_O_2_ measured was significantly different between experimental runs, and independent of particle loading. This was likely because the effect of increasing the reactive surface was twofold: (i) an increase in the formation of H_2_O_2_, and (ii) an increase in the release of dissolved Fe^2+^, accelerating the decomposition of H_2_O_2_ by the Fenton reaction (Fig. 7). We plotted H_2_O_2_ and Fe^2+^evolution as predicted by the model for a set of different values of pyrite reactive surface area (in Fig. 7a). As a result of this coupling effect, higher values of reactive surface area tend to increase the amount of secondary ROS (i.e., OH^**·**^/O^−^, HO_2_^**·**^/O_2_^**·**−^, O_2_) rather than stabilize the presence of H_2_O_2_ in solution (in Fig. 7b)^2^,^48^.

## Discussion

Aqueous suspensions of pyrite form H_2_O_2_, in the presence and absence of dissolved O_2_, following a generation-decay trend (Fig. 1). The asymmetric shape observed in the experimental curves indicated that the apparent rate of H_2_O_2_ generation was significantly faster than the apparent H_2_O_2_ degradation. Previous studies^22^,^25^ have suggested that in the presence of dissolved O_2_, pyrite slurries form H_2_O_2_ by an electron transfer between the ferrous iron defect sites (Fe^2+^−S) and the adsorbed O_2_ through a Habber-Weiss reaction, involving the O_2_^**·**−^ radical formation (equations 1 and 2). This hypothesis is consistent with the observation that there is an inverse relationship between H_2_O_2_ and O_2_ at the beginning of the oxic experiment (Fig. 2a). The mechanism of H_2_O_2_ formation in anoxic conditions remains more controversial. Some studies have suggested that in absence of O_2_ the formation of H_2_O_2_ is driven by the oxidation of adsorbed H_2_O catalyzed by the pyrite surface^4^,^19^,^24^,^26^, whereas other studies have considered that this reaction is unlikely due to energetic considerations^15^,^25^. A possible reconciliation comes from considering the presence of ≡Fe^3+^dangling bonds generated from the cleavage of S-S bonds. Briefly, the rupture of S-S bonds generate S^−^ species which are highly instable and, to compensate its charge disequilibrium, donate one electron to the nearest iron atom by the autoredox reaction^26^,^31^,^32^,^49^,^50^,^51^,^52^,^53^,^54^:


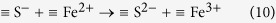


These dangling bonds could decrease the energy requirements for the chemisorption of H_2_O molecules, some authors even talk about “ferryl” iron dangling bonds ≡Fe^4+^ 55, leading to H_2_O splitting into H^+^and OH^**·**^, with the subsequent formation of H_2_O_2_, as described by^19^ equations 3 and 4. Our experiments showed that H_2_O_2_ was generated in the absence of both dissolved O_2_ and light (Fig. 1b). Besides, the estimated ratio of S/Fe (<2) in unreacted pyrite particles (Supplementary Fig. S8) indicated the presence of S vacancies.

Experiments with pyrite slurries under both oxic and anoxic conditions showed a sudden drop of pH during the first hours (Supplementary Fig. S2). This increase of H^+^concentration together with the progressive accumulation of H_2_O_2_ and Fe^2+^in solution is expected to trigger the Fenton reaction. UV-Vis spectroscopy confirmed the presence of Fe^3+^-complexes during anoxic pyrite dissolution (Fig. 3a) whose maximum wavelengths were compatible with the following reaction^41^:


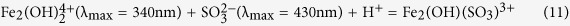


The detection of both Fe^3+^-complexes and OH^**·**^ in solution (Fig. 3) indicated that the Fenton reaction occurred even in the absence of dissolved O_2_, supporting the idea that the suite of Fenton reactions conditioned the decay period of H_2_O_2_ curves. Additionally, the formation of OH^**·**^ concomitantly with the formation of H_2_O_2_ from the start-up of the experiment under anoxic conditions (Fig. 3b) together with the splitting of the cathodic peak associated with the nearly spontaneous iron reduction (Supplementary Fig. S3), suggest that ≡Fe^3+^dangling bonds, actually catalyze the H_2_O oxidation by one single electron, forming OH^**·**^ as described by equation 3.

In principle, the overall process of aqueous pyrite oxidation -under oxic conditions- involves only O_2 (g)_ consumption, according to:


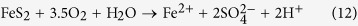


However, considering a free radical mechanism, a simultaneous uptake and release of dissolved O_2_ it to be expected, because it can be both a product and a reactant. In oxic experiments, O_2_ concentration rapidly dropped and then reached a more constant value (Fig. 2a). Since the amount of Fe^2+^in solution is presumably low at these early stages, the consumption of O_2_ during this period could be attributed to Fe^2+^oxidation at surface sites to form the superoxide anion (O_2_^**·**−^) and to the subsequent production of H_2_O_2_ (equations 1 and 2). An interesting result was the formation of O_2 (aq)_ as a by-product in anoxic experiments (Fig. 2b and Supplementary Fig. S1b). Although O_2_ is not a direct product in the equation 3, since H_2_O_2_ is formed, the formation of O_2_ can occur via several pathways such as the Fenton-like reaction, the “catalase-like reaction”^25^ or via non-radical disproportionation of H_2_O_2._

XPS and HRTEM results reported herein showed the formation of Fe^3+^−O patches over pyrite surfaces. The implication of these Fe^3+^−patches during aqueous pyrite oxidation is not clear. Some studies argued that the electron cycling of Fe^2+^and Fe^3+^between unoxidized and oxidized areas favors the electron transfer from the surface of the pyrite to molecular O_2_, increasing the oxidation rate of pyrite^56^. However, this mechanism is based on atmospheric oxidation of pyrite and does not explain the formation of H_2_O_2_. In contrast, other studies have suggested that the formation of these Fe^3+^−patches interrupts the redox cycling of Fe^2+^/Fe^3+^, thereby inhibiting the formation of H_2_O_2_ and decreasing the rate of pyrite dissolution^25^. Our cycling experiments showed that most of the defect sites of the pyrite microparticles remained active at the end of the experiment, even when H_2_O_2_ was no longer observable in solution (Fig. 4). Moreover, it is expected that oxidized patches will desorb at low pH during the pyrite dissolution process. HRTEM images showed an appreciable increment of the pyrite alteration layer (Supplementary Fig. S9), suggesting that these Fe^3+^−patches failed to completely block the surface renewal during pyrite dissolution.

Based on our experimental results, we propose a model that explains the generation-decay trend of H_2_O_2_ in terms of a kinetic competition between (1) the formation of H_2_O_2_ by the self-oxidation of iron-sulfur cluster defect sites; and, (2) the degradation of H_2_O_2_ by the Fenton reaction triggered by pyrite dissolution. Accordingly, the evolution of H_2_O_2_ in solution can be summarized as follows:


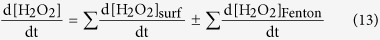


At the beginning of the model run (t ≈ 0), the amount of H_2_O_2_ in solution was controlled by the surface reaction and the second term tended toward zero. However, when the Fenton reaction started, H_2_O_2_ was progressively degraded to secondary ROS, in solution (i.e., OH^**·**^, HO_2_^**·**^/O_2_^**·**−^, O_2_). At the end of the model run the amount of H_2_O_2_ remained constant within a value close to zero and equation 13, became:


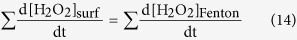


The model allowed us to estimate the rate constants of H_2_O_2_ formation under both oxic and anoxic conditions (Table 1). Peak production of H_2_O_2_ was shifted towards longer times in anoxic conditions (Supplementary Fig. S10), pointing to slower oxidation kinetics of pyrite in the absence of O_2_, a result also supported by our XPS analysis (Supplementary Figs S4 and 5) and by the sulfate and iron released by pyrite dissolution (Supplementary Fig. S11). When compared with experimental data, the model reproduces qualitatively well the observed trends for pH, O_2_, Fe_total_ and SO_4_^2−^, as these parameters are calculated using the overall equations of pyrite dissolution^17^,^57^,^58^ (Fig. 6a–b). An additional feature of the model is that provides a way to reconcile the classical dissolution approach with the free radical assumption, opening a new pathway to analyze the flux of secondary ROS resulting from the degradation of H_2_O_2_ through the Fenton reaction (Fig. 6c–d). OH^**·**^ radicals were the first species produced, rapidly decaying to HO_2_^**·**^/O_2_^**·**−^. These ROS species counterbalances the decrease in pH and promoted the so-called “Fenton like” reaction, which resulted in the formation of O_2_(g). Fe^2+^regenerated through this sequence, and also through the dissolution of pyrite, makes the Fenton reaction more efficient^59^. Therefore, the disappearance of H_2_O_2_ in solution was likely due to a fast transformation into ROS, catalyzed by dissolved Fe^2+^, rather than by the cessation of the generation mechanism itself. This result is consistent with the continuous production of H_2_O_2_ and OH^**·**^^23^,^26^,^60^ during pyrite dissolution. We hypothesize that as a result of the progressive acidification of the solution together with iron recycling by the Fenton reaction, the abiotic dissolution of pyrite microparticles can be considered as a natural and auto-catalytic Fenton reagent, useful to understanding long-term oxidation processes even in oxygen limited environments.

## Methods

### Sample preparation and characterization

Natural pyrite samples (Logroño, Spain) were ground to obtain particles with average diameter of 1.4 μm, Laser Diffraction Particle Size Analyzer (LS13320) and BET (Brunauer – Emmett – Teller) surface area of 1.46 m^2^/g (multi-point N_2_ adsorption). Prior to use, freshly ground pyrite microparticles were washed by sonication in ethanol (96%) and hydrochloric acid (HCl 0.25 M), to remove organics and oxide surface coatings. Samples were then rinsed with deoxygenated deionized water (MilliQ) and dried in a low vacuum chamber purged with nitrogen (N_2_) until used. Minor and trace elements in the acid washed samples were evaluated by scanning electron microscopy (SEM) using X-ray mapping (XRM) (Supplementary Fig. S8). X-ray diffraction (XRD) was used to assess the presence of secondary phases and to characterize the degree of structural disorder in the samples using the Rietveld method (Supplementary Fig. S12).

### Batch kinetic experiments

The kinetics of H_2_O_2_ formation and degradation on pyrite slurries was investigated in batch reactors utilizing amperometric sensors for H_2_O_2_ (ISO-HPO-100, World Precision Instruments, Inc.) and dissolved O_2_ (Unisense DK), and a glass electrode for pH determination (Vernier FPH-BTA). Batch reactors were designed to fit with microsensors, spectroscopic probes, ports for pyrite inlet under N_2_ atmosphere, and valves. The valves allowed fluid circulation and solution sampling in a closed system configuration.

The production of ferric iron (Fe^3+^) and hydroxyl radical (OH^**·**^) species by pyrite slurries in absence of dissolved O_2_ were measured using UV-vis spectroscopy by monitoring absorption bands at specific wavelengths. To prevent data masking due to particle absorption, the pyrite microparticles were deposited onto silicone strips as a thin film adhered to the inner reactor walls. Spectroscopic data were collected with a fiber optic UV-Vis spectrometer (Black-comet, Stellarnet or USB4000, Ocean Optics) and acquired with the SpectraWiz^®^ or loggerpro3 codes. In addition, total dissolved iron and sulfate released during pyrite dissolution were measured at different time intervals using inductively coupled plasma mass spectrometry (ICP-MS) and ion chromatography (IC).

All experiments were made under continuous magnetic stirring (500 rpm), at room temperature (T° ∼ 22 °C) and in the dark, unless other conditions are specified. Due to the autocatalytic nature of Fenton chain reactions, kinetic experiments were carried out in unbuffered distilled water. Further details of the batch reactors, electrochemical sensors, experimental procedures and test analysis are given as supplementary material (Supplementary Figs S13–18).

### Surface analysis

Cyclic Voltammetry (CV) was employed as an additional way to assess the sequence of redox reactions involving free radicals during H_2_O adsorption at pyrite interface. CV was performed using Pt/Pyrite-Np’s/ Nafion©/ electrodes in N_2_ purged solutions vs Ag/Cl 3 M KCl. A detailed description of the experimental set-up is given as Supplementary Material. X-ray photoelectron spectroscopy (XPS) was used to analyze the surface oxidation states of (001) faces of pyrite (single-crystal, ~1 cm^2^ × 2 mm) after aqueous reaction in oxic and anoxic conditions. Platelets parallel to (001) faces were cut (1 cm^2^ × 2 mm) and cleaned following the same procedure described above and allowed to react with water in oxic and anoxic conditions. Samples were dried and stored in N_2_-filled tubes until introduction into the XPS vacuum chamber. The oxidation states were analyzed at three different stages of the dissolution process: (t_1_) unreacted sample; the pyrite crystal was acid-washed to generate oxide-free surfaces by removing the normal contaminants, carbon, nitrogen, and oxygen (C, N, O) due to atmospheric oxidation; (t_2_): sample after 22 hours immersed in oxic water. (t_3_): sample after 22 hours immersed in anoxic water. Prior to this stepped analysis, the XPS spectra of a pyrite surface was analyzed after argon ion (Ar^+^) sputtering to verify the formation of Fe^3+^and S^2−^ surface species as occurs during mechanical fracture by preferential sulfur removal. XPS Spectra were collected from a take-off angle of 90° relative to the sample surface in a Thermo Scientific K-Alpha ESCA analyzer using monochromatic Al Kα (1486.6 eV) radiation and pass energies of 100 eV and 20 eV for survey spectra and narrow region spectra, respectively. Spectra were aligned by setting the C1s peak to a binding energy of 285 eV^61^. Deconvolution and fitting of experimental data were done with the XPSpeak4.2 software (http://www.phy.cuhk.edu.hk/~surface/XPSPEAK/). The Shirley method was used for background subtraction and the binding energies of the species identified were assigned using values taken from literature (Supplementary Tables S2 and 3).

High resolution Electron Transmission Microscopy (HTREM) analysis were performed to identify nano-domains of secondary oxidation products at the pyrite interface. HTREM images were acquired on a JEOL JEM- 3011 microscope with accelerating voltage of 200 kV using a Gatan Ultrascan 1000 CCD camera and Digital Microgrograph software. Data processing was performed with the GADDS and image-J codes. Pyrite lamellas were prepared using a focused ion beam (FIB) with a high resolution JEOL JSM-6700 f.

### Kinetic model

The model was run using the computer code Copasi 4.8 (COmplex PAthway SImulator)^62^. We assume that the experimental trend of H_2_O_2_, in the presence and absence of O_2_ (g), is shaped by three main processes: (i) the rate of H_2_O_2_ generation by the iron defect sites on the surface of pyrite particles; (ii) the production rates of Fe^2+^and SO_4_^2−^ by pyrite dissolution; and, (iii) the kinetics of H_2_O_2_ degradation by the Fenton reaction. Fits to the experimental curves that describe the evolution of H_2_O_2_ were performed by using the Marquardt algorithm employing as adjustable parameters the rate constants of the H_2_O_2_ formation. A detailed explanation of the modeling set-up is included in the supplementary material.

## Additional Information

**How to cite this article:** Gil-Lozano, C. *et al*. Quantifying Fenton reaction pathways driven by self-generated H_2_O_2_ on pyrite surfaces. *Sci. Rep.* **7**, 43703; doi: 10.1038/srep43703 (2017).

**Publisher's note:** Springer Nature remains neutral with regard to jurisdictional claims in published maps and institutional affiliations.

## Supplementary Material

Supplementary Material

## Figures and Tables

**Table 1:** Rate constants obtained by fitting experimental data.

Rate constants	Oxic conditions	Anoxic conditions
*k*_oxic_ (mol/m^2^s)	10^−3.58^	10^−3.70^
*k*_anoxic_ (mol/m^2^s)	10^−8.25^	10^−8.14^
*k_pyr_	by O_2_ 10^−7.86^ (mol/ m^2^ s)	by H_2_O_2_ 10^−6.03^ (L/ m^2^ s)

^*^The rate of pyrite oxidation by H_2_O_2_ was used as an additional adjustable parameter based on the expression given by McKibben and Barnes (1987) whereas the oxidation rate by O_2_ was used as a fixed parameter (Liu *et al*. 2008).

## References

[b1] Puthussery J., Seefeld S., Berry N., Gibbs M. & Law M. Colloidal Iron Pyrite (FeS2) Nanocrystal Inks for Thin-Film Photovoltaics. Journal of the American Chemical Society 133, 716–719, doi: 10.1021/ja1096368 (2010). 21175173

[b2] Bae S., Kim D. & Lee W. Degradation of diclofenac by pyrite catalyzed Fenton oxidation. Applied Catalysis B: Environmental 134–135, 93–102, doi: 10.1016/j.apcatb.2012.12.031 (2013).

[b3] Gil-Lozano C., Losa-Adams E., Davila F. A. & Gago-Duport L. Pyrite nanoparticles as a Fenton-like reagent for *in situ* remediation of organic pollutants. Beilstein journal of nanotechnology 5, 855–864, doi: 10.3762/bjnano.5.97 (2014). 24991522 PMC4077362

[b4] Wang W., Qu Y., Yang B., Liu X. & Su W. Lactate oxidation in pyrite suspension: A Fenton-like process *in situ* generating H2O2. Chemosphere 86, 376–382, doi: 10.1016/j.chemosphere.2011.10.026 (2012). 22099540

[b5] Che H. & Lee W. Selective redox degradation of chlorinated aliphatic compounds by Fenton reaction in pyrite suspension. Chemosphere 82, 1103–1108, doi: 10.1016/j.chemosphere.2010.12.002 (2011). 21186044

[b6] Choi K., Bae S. & Lee W. Degradation of pyrene in cetylpyridinium chloride-aided soil washing wastewater by pyrite Fenton reaction. Chemical Engineering Journal 249, 34–41, doi: 10.1016/j.cej.2014.03.090 (2014).

[b7] Choi K., Bae S. & Lee W. Degradation of off-gas toluene in continuous pyrite Fenton system. Journal of Hazardous Materials 280, 31–37, doi: 10.1016/j.jhazmat.2014.07.054 (2014). 25125037

[b8] Chandra A. P. & Gerson A. R. The mechanisms of pyrite oxidation and leaching: A fundamental perspective. Surface Science Reports 65, 293–315, doi: 10.1016/j.surfrep.2010.08.003 (2010).

[b9] Descostes M., Vitorge P. & Beaucaire C. Pyrite dissolution in acidic media. Geochimica et Cosmochimica Acta 68, 4559–4569, doi: 10.1016/j.gca.2004.04.012 (2004).

[b10] Druschel G. & Borda M. Comment on “Pyrite dissolution in acidic media” by M. Descostes, P. Vitorge, and C. Beaucaire. Geochimica et Cosmochimica Acta 70, 5246–5250, doi: 10.1016/j.gca.2005.07.023 (2006).

[b11] Heidel C. & Tichomirowa M. The isotopic composition of sulfate from anaerobic and low oxygen pyrite oxidation experiments with ferric iron — New insights into oxidation mechanisms. Chemical Geology 281, 305–316, doi: 10.1016/j.chemgeo.2010.12.017 (2011).

[b12] Lowson R. T. Aqueous oxidation of pyrite by molecular oxygen. Chemical Reviews 82, 461–497, doi: 10.1021/cr00051a001 (1982).

[b13] Moses C. O., Kirk Nordstrom D., Herman J. S. & Mills A. L. Aqueous pyrite oxidation by dissolved oxygen and by ferric iron. Geochimica et Cosmochimica Acta 51, 1561–1571, doi: 10.1016/0016-7037(87)90337-1 (1987).

[b14] Rimstidt J. D. & Vaughan D. J. Pyrite oxidation: a state-of-the-art assessment of the reaction mechanism. Geochimica et Cosmochimica Acta 67, 873–880, doi: 10.1016/s0016-7037(02)01165-1 (2003).

[b15] Rosso K. M., Becker U. & Hochella M. F. Jr The interaction of pyrite {100} surfaces with O2 and H2O: Fundamental oxidation mechanisms. American Mineralogist 84, 1549–1561, doi: 10.2138/am-1999-1008 (1999).

[b16] Singer P. C. & Stumm W. Acidic mine drainage: The rate-determining step. Science 167, 1121–1123, doi: 10.1126/science.167.3921.1121 (1970). 17829406

[b17] Williamson M. A. & Rimstidt J. D. The kinetics and electrochemical rate-determining step of aqueous pyrite oxidation. Geochimica et Cosmochimica Acta 58, 5443–5454, doi: 10.1016/0016-7037(94)90241-0 (1994).

[b18] Borda M. J., Elsetinow A. R., Schoonen M. A. & Strongin D. R. Pyrite-Induced Hydrogen Peroxide Formation as a Driving Force in the Evolution of Photosynthetic Organisms on an Early Earth. Astrobiology 1, 283–288, doi: 10.1089/15311070152757474 (2001). 12448991

[b19] Borda M. J., Elsetinow A. R., Strongin D. R. & Schoonen M. A. A mechanism for the production of hydroxyl radical at surface defect sites on pyrite. Geochimica et Cosmochimica Acta 67, 935–939, doi: 10.1016/s0016-7037(02)01222-x (2003).

[b20] Cohn C. A., Borda M. J. & Schoonen M. A. RNA decomposition by pyrite-induced radicals and possible role of lipids during the emergence of life. Earth and Planetary Science Letters 225, 271–278, doi: 10.1016/j.epsl.2004.07.007 (2004).

[b21] Cohn C. A., Pak A., Strongin D. R. & Schoonen M. A. Quantifying hydrogen peroxide in iron-containing solutions using leuco crystal violet. Geochemical Transactions 6, 47, doi: 10.1186/1467-4866-6-47 (2005). PMC1475790 35412761

[b22] Cohn C. A. . Pyrite-induced hydroxyl radical formation and its effect on nucleic acids. Geochemical Transactions 7, 11, doi: 10.1186/1467-4866-7-3 (2006). 16759350 PMC1523326

[b23] Cohn C., Fisher S., Brownawell B. & Schoonen M. Adenine oxidation by pyrite-generated hydroxyl radicals. Geochemical Transactions 11, 8, doi: 10.1186/1467-4866-11-2 (2010). PMC2873965 20420694

[b24] Javadi N. A. & Hanumantha R. K. Formation of hydrogen peroxide by sulphide minerals. Hydrometallurgy 141, 82–88, doi: 10.1016/j.hydromet.2013.10.011 (2014).

[b25] Schoonen M. A. A., Harrington A. D., Laffers R. & Strongin D. R. Role of hydrogen peroxide and hydroxyl radical in pyrite oxidation by molecular oxygen. Geochimica et Cosmochimica Acta 74, 4971–4987, doi: 10.1016/j.gca.2010.05.028 (2010).

[b26] Zhang P., Yuan S. & Liao P. Mechanisms of hydroxyl radical production from abiotic oxidation of pyrite under acidic conditions. Geochimica et Cosmochimica Acta 172, 444–457, doi: 10.1016/j.gca.2015.10.015 (2016).

[b27] Burns R. G. & Fisher D. S. Iron-sulfur mineralogy of Mars: Magmatic evolution and chemical weathering products. Journal of Geophysical Research: Solid Earth 95, 14415–14421, doi: 10.1029/JB095iB09p14415 (1990).

[b28] Davila A. F. . Subsurface formation of oxidants on Mars and implications for the preservation of organic biosignatures. Earth and Planetary Science Letters 272, 456–463, doi: 10.1016/j.epsl.2008.05.015 (2008).

[b29] Eggleston C. M., Stern J. R., Strellis T. M. & Parkinson B. A. A natural photoelectrochemical cell for water splitting: Implications for early Earth and Mars. American Mineralogist 97, 1804–1807, doi: 10.2138/am.2012.4211 (2012).

[b30] Zolotov M. Y. & Shock E. L. Formation of jarosite-bearing deposits through aqueous oxidation of pyrite at Meridiani Planum, Mars. Geophys. Res. Lett. 32, L21203, doi: 10.1029/2005gl024253 (2005).

[b31] Nesbitt H. W., Bancroft G. M., Pratt A. R. & Scaini M. J. Sulfur and iron surface states on fractured pyrite surfaces. American Mineralogist 83, 1067–1076, doi: 10.2138/am-1998-9-1015 (1998).

[b32] Schaufuß A. G. . Reactivity of surface chemical states on fractured pyrite. Surface Science 411, 321–328, doi: 10.1016/s0039-6028(98)00355-0 (1998).

[b33] Murphy R. & Strongin D. R. Surface reactivity of pyrite and related sulfides. Surface Science Reports 64, 1–45, doi: 10.1016/j.surfrep.2008.09.002 (2009).

[b34] Krishnamoorthy A., Herbert F. W., Yip S., Van Vliet K. J., B. Yildiz, Herbert, Sidney F. W., , Krystyn J. V. V. & Bilge Y. Electronic states of intrinsic surface and bulk vacancies in FeS2. Journal of Physics: Condensed Matter 25, 045004, doi: 10.1088/0953-8984/25/4/045004 (2012). 23220862

[b35] Wang J., Chen D., Yan D., Wei H. & Xiang L. Evolution from an anoxic to oxic deep ocean during the Ediacaran–Cambrian transition and implications for bioradiation. Chemical Geology 306–307, 129–138, doi: 10.1016/j.chemgeo.2012.03.005 (2012).

[b36] Guevremont J. M., Elseinow A. R., Strongin D. R., Bebie J. & Schoonen M. A. A. Structure sensitivity of pyrite oxidation; comparison of the (100) and (111) planes. American Mineralogist 83, 1353–1356, doi: 10.2138/am-1998-11-1225 (1998).

[b37] Guevremont J. M., Strongin D. R. & Schoonen M. A. A. Thermal chemistry of H2S and H2O on the (100) plane of pyrite: unique reactivity of defect sites Vol. 83 (Mineralogical Society of America, 1998).

[b38] de Leeuw N. H., Parker S. C., Sithole H. M. & Ngoepe P. E. Modeling the Surface Structure and Reactivity of Pyrite: Introducing a Potential Model for FeS2. The Journal of Physical Chemistry B 104, 7969–7976, doi: 10.1021/jp0009498 (2000).

[b39] Fischer C., Kurganskaya I., Schäfer T. & Lüttge A. Variability of crystal surface reactivity: What do we know? Applied Geochemistry 43, 132–157, doi: 10.1016/j.apgeochem.2014.02.002 (2014).

[b40] Feng W. & Nansheng D. Photochemistry of hydrolytic iron (III) species and photoinduced degradation of organic compounds. A minireview. Chemosphere 41, 1137–1147, doi: 10.1016/s0045-6535(00)00024-2 (2000). 10901238

[b41] Lente G. Reactions of the iron(III) hydroxo dimer with inorganic ligands, University of Debrecen, (2001).

[b42] Ma J., Weng D., Wu X., Si Z. & Wu Z. Highly dispersed iron species created on alkali-treated zeolite for ammonia SCR. Progress in Natural Science: Materials International 23, 493–500, doi: 10.1016/j.pnsc.2013.08.005 (2013).

[b43] Stefánsson A. Iron(III) Hydrolysis and Solubility at 25 °C. Environmental Science & Technology 41, 6117–6123, doi: 10.1021/es070174h (2007). 17937290

[b44] Fan H.-J. . Degradation pathways of crystal violet by Fenton and Fenton-like systems: Condition optimization and intermediate separation and identification. Journal of Hazardous Materials 171, 1032–1044, doi: 10.1016/j.jhazmat.2009.06.117 (2009). 19604632

[b45] Banfield J. F., Welch S. A., Zhang H. & Ebert T. T. & Penn, R. L. Aggregation-Based Crystal Growth and Microstructure Development in Natural Iron Oxyhydroxide Biomineralization Products. Science 289, 751–754, doi: 10.1126/science.289.5480.751 (2000). 10926531

[b46] Drits V. A., Sakharov B. A., Salyn A. L. & Manceau A. Structural model for ferrihydrite. Clay Minerals 28, 185–207, doi: 10.1180/claymin.1993.028.2.02 (1993).

[b47] Das S., Hendry M. J. & Essilfie-Dughan J. Transformation of Two-Line Ferrihydrite to Goethite and Hematite as a Function of pH and Temperature. Environmental Science & Technology 45, 268–275, doi: 10.1021/es101903y (2011). 21128633

[b48] Che H., Bae S. & Lee W. Degradation of trichloroethylene by Fenton reaction in pyrite suspension. Journal of Hazardous Materials 185, 1355–1361, doi: 10.1016/j.jhazmat.2010.10.055 (2011). 21071138

[b49] Nesbitt H. W. . Synchrotron XPS evidence for Fe^2+^−S and Fe^3+^−S surface species on pyrite fracture-surfaces, and their 3D electronic states. American Mineralogist 85, 850–857, doi: 10.2138/am-2000-5-628 (2000).

[b50] Leiro J. A., Mattila S. S. & Laajalehto K. XPS study of the sulphur 2p spectra of pyrite. Surface Science 547, 157–161, doi: 10.1016/j.susc.2003.09.033 (2003).

[b51] Andersson K. . Experimental and theoretical characterization of the structure of defects at the pyrite FeS2 (100) surface. Physical Review B 70, 195404, doi: 10.1103/PhysRevB.70.195404 (2004).

[b52] Zhang Y. N., Hu J., Law M. & Wu R. Q. Effect of surface stoichiometry on the band gap of the pyrite FeS2 (100) surface. Physical Review B 85, 085314, doi: 10.1103/PhysRevB.85.085314 (2012).

[b53] Herbert F. W., Krishnamoorthy A., Van Vliet K. J. & Yildiz B. Quantification of electronic band gap and surface states on FeS2(100). Surface Science 618, 53–61, doi: 10.1016/j.susc.2013.08.014 (2013).

[b54] Sanchez-Arenillas M. & Mateo-Marti E. Pyrite surface environment drives molecular adsorption: cystine on pyrite(100) investigated by X-ray photoemission spectroscopy and low energy electron diffraction. Physical Chemistry Chemical Physics 18, 27219–27225, doi: 10.1039/C6CP03760G (2016). 27711447

[b55] Stirling A., Bernasconi M. & Parrinello M. Defective pyrite (100) surface: An ab initio study. Physical Review B 75, 8, doi: 10.1103/PhysRevB.75.165406 (2007).

[b56] Eggleston C. M., Ehrhardt J.-J. & Stumm W. Surface structural controls on pyrite oxidation kinetics; an XPS-UPS, STM, and modeling study. American Mineralogist 81, 1036–1056, doi: 10.2138/am-1996-9-1002 (1996).

[b57] Liu R., Wolfe A., Dzombak D., Stewart B. & Capo R. Comparison of dissolution under oxic acid drainage conditions for eight sedimentary and hydrothermal pyrite samples. Environ Geol 56, 171–182, doi: 10.1007/s00254-007-1149-0 (2008).

[b58] McKibben M. A. & Barnes H. L. Oxidation of pyrite in low temperature acidic solutions: Rate laws and surface textures. Geochimica et Cosmochimica Acta 50, 1509–1520, doi: 10.1016/0016-7037(86)90325-X (1986).

[b59] Duesterberg C. K., Mylon S. E. & Waite T. D. pH Effects on Iron-Catalyzed Oxidation using Fenton’s Reagent. Environmental Science & Technology 42, 8522–8527, doi: 10.1021/es801720d (2008). 19068842

[b60] Fisher S., Schoonen M. & Brownawell B. Phenylalanine as a hydroxyl radical-specific probe in pyrite slurries. Geochemical Transactions 13, 1–18, doi: 10.1186/1467-4866-13-3 (2012). 22313632 PMC3348026

[b61] Briggs D. & Seah M. P. Practical Surface Analysis: Auger and X-ray photoelectron spectroscopy. 2 nd edn, Vol. 1 657 (John Wiley & Sons, 1990).

[b62] Hoops S. . COPASI—a COmplex PAthway SImulator. Bioinformatics 22, 3067–3074, doi: 10.1093/bioinformatics/btl485 (2006). 17032683

