# Supplementary material for: Quantifying Fenton reaction pathways driven by self-generated H2O2 on pyrite surfaces
Source: Sci Rep. 2017 Mar 6;7:43703. doi: 10.1038/srep43703 (PMC5337962; doi:10.1038/srep43703)
Supplement: Supplementary Material [file srep43703-s1.pdf]

Supplementary material

Quantifying Fenton reaction pathways driven by H_2O_2 self-generated on pyrite surfaces

C. Gil-Lozano[1]*, A.F. Davila[2], E. Losa-Adams[1, 3], A.G. Fairén[1, 4] and L.Gago-Duport[3]

[1]Centro de Astrobiología (CSIC-INTA), 28850 Torrejón de Ardoz, Madrid, Spain.

*cgil@inta.cab-csic.es

[2]Carl Sagan Center at the SETI Institute, 189 Bernardo Avenue, Suite 100, Mountain View, CA 94043, USA

[3]Departamento de Geociencias Marinas, Universidad de Vigo, Lagoas Marcosende, 36310-Vigo, Spain.

[4]Department of Astronomy, Cornell University, Ithaca, 14853 NY, USA

Supplementary data includes 18 figures and 3 tables.

1. Figures.

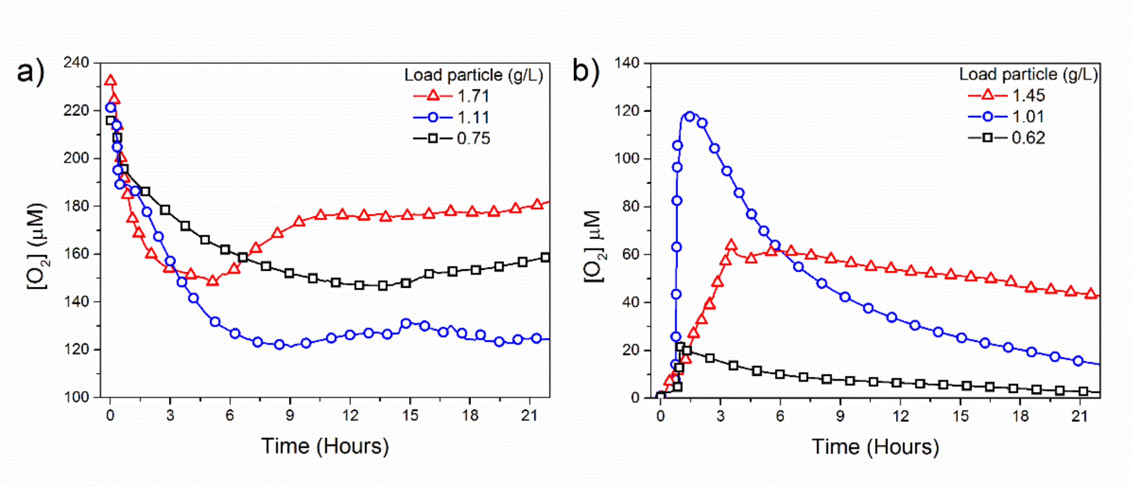

Figure S.1. O_2 evolution from pyrite slurries in unbuffered water -at different particle loading (inset)- under a) oxic and b) anoxic conditions. O_2 evolution shows opposite trends under oxic (asymptotic decrease followed by a slight increase and a steady stable period at the end of the experiment) and anoxic conditions (initial increase followed by an asymptotic decrease).

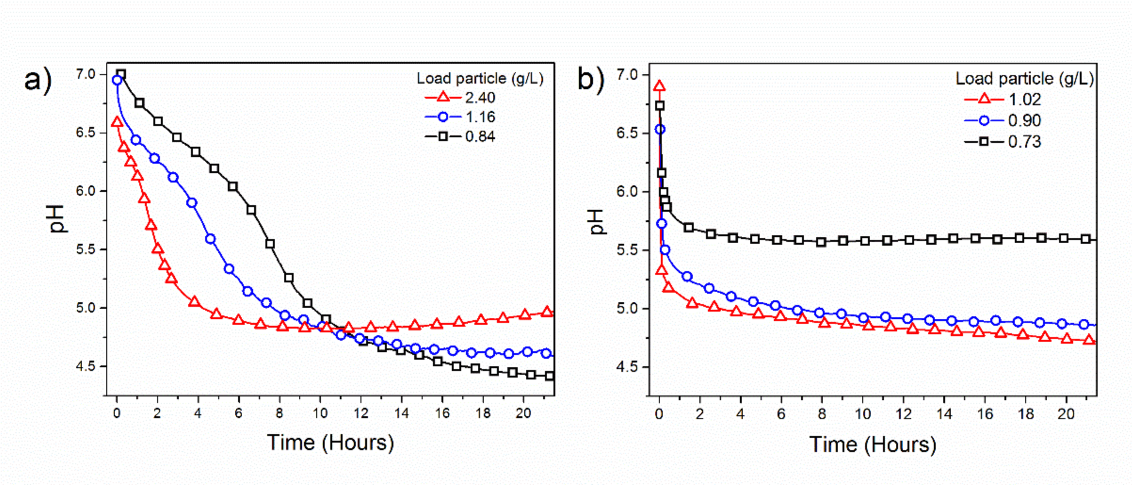

Figure S.2. pH evolution in unbuffered pyrite slurries -with different particle loading (inset)- under a) oxic and b) anoxic conditions. In both oxic and anoxic conditions, pH drops rapidly towards a nearly constant value (approximately 2 to 3 pH units lower than initial pH values). As expected, samples with high pyrite loading reached that value faster than samples with low loading. The decrease was more pronounced under anoxic than under oxic conditions (2 hours vs 10 hours, respectively).

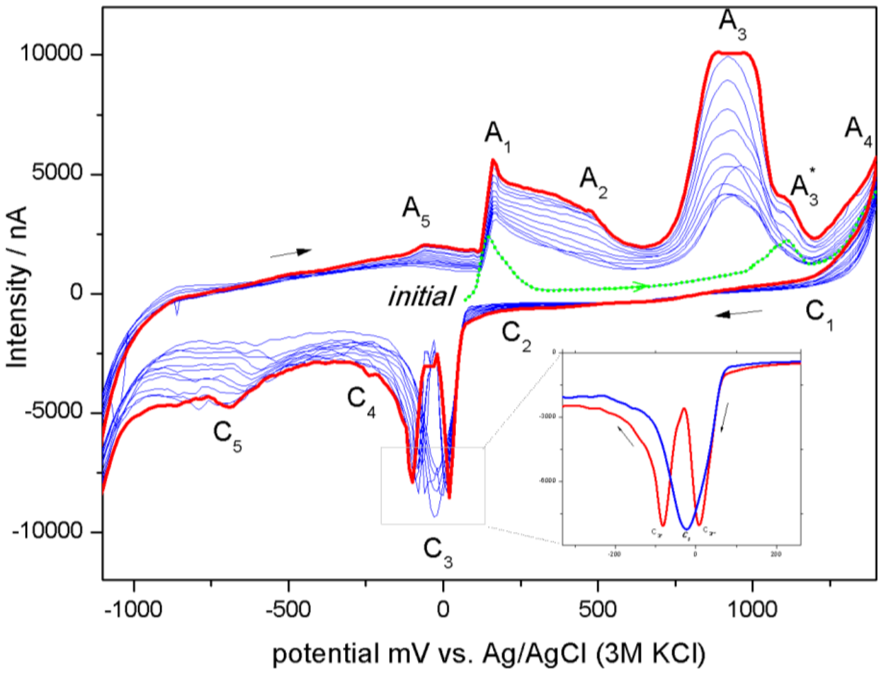

Figure S.3. Cyclic voltammetry of Pt/PyriteNp´s/Nafion/ electrodes in PBS (pH 7.2), scan rate 10 mV/s. The voltammograms were initiated from the open circuit potential on a positive-going direction, and then, switched at 1400 mV Ag/AgCl to negative-going direction. The first anodic peak centered at 0.31V NHE, correspond to the iron oxidation reaction (A_1, $\equiv Fe^{2+} \rightarrow \equiv Fe^{3+} + \bar{e}$). When increasing the potential until the anodic switching value, where H_2O oxidation takes place by a four electron mechanism ($2H_2O \rightarrow O_2 + 4e^- + 4H^+$, 1.23V NHE), we also found two intermediate irreversible peaks (A_2, 0.61V NHE and A_3, 1.06V NHE), which may be associated with H_2O oxidation by one single electron transfer (A_2, $H_2O \rightarrow OH^{\bullet} + H^+ + \bar{e}$) and by two electron transfer (A_3, $2H_2O \rightarrow H_2O_2 + 2\bar{e} + 2H^+$), respectively. In the cathodic counterpart, the peak assigned to iron reduction takes place at low potential value (C3, $\equiv Fe^{3+} + \bar{e} \rightarrow \equiv Fe^{2+}$, 0.2V NHE) and is split into two minima, (C3', C3'') at 0.1 V NHE and 0.2 V NHE, respectively. This suggested that a fraction of the previously oxidized iron, probably those associated with iron dangling bonds, is reduced in a nearly spontaneous manner, triggering the formation of $OH^{\bullet}$ from the oxidation of the adsorbed H_2O by one electron transfer, mentioned above.

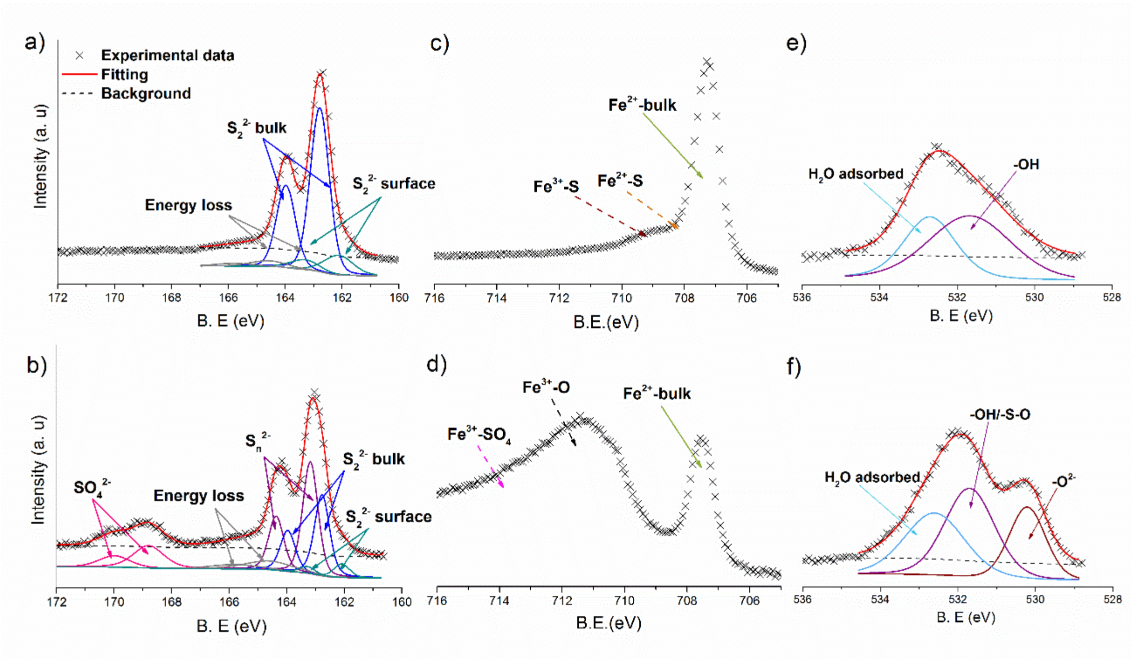

Figure S. 4. a) S2p orbital of the clean sample (t1), showing the S_2^{2-}-surface and S_2^{2-}-bulk contributions. We also identified a peak in the region of 164.6 eV, previously associated with a bulk energy loss feature [1,2], whose contribution remained nearly constant after pyrite aqueous reaction. b) S2p orbital after 22h of aqueous reaction under oxic conditions (t2), showing a shift to higher binding energies, which indicates an increment in the sulfur oxidation states. The peaks assigned to S_n^{2-} (163.2 eV) and SO_4^{2-} (168.8 eV) were also observed. c) Fe2p$_{3/2}$ orbital of the clean sample (t1). Overall, the spectrum shows a well-defined peak ascribed to Fe^{2+} bulk contribution and a small tail probably associated with iron surface species. d) Fe2p$_{3/2}$ orbital after 22h of aqueous reaction under oxic conditions (t2). A broad and asymmetrical peak appeared near to the binding energy range characteristic of iron oxides/hydroxides (Fe^{3+}-O, 711 eV) and ferric sulfates (Fe^{3+}-SO4, 713.3 eV), according to the NIST database values. e) O1s orbital of the clean sample (t1). We detected a peak assigned to H_2O contribution (532. eV), and a second peak centered at 531.7 eV that can be associated with either hydroxyl or sulfur oxidation products (-OH/S-O), as the binding energies of both species overlap in this range. However, the S2p spectra did not show S-O species at t1. f) O1s orbital after 22h of aqueous reaction under oxic conditions (t2). The spectrum shows an increment of the peak's asymmetry due to a new oxygen contribution at 530.2 eV, ascribed with iron oxides (O^{2-}), also observed in the Fe2p$_{3/2}$ spectrum.

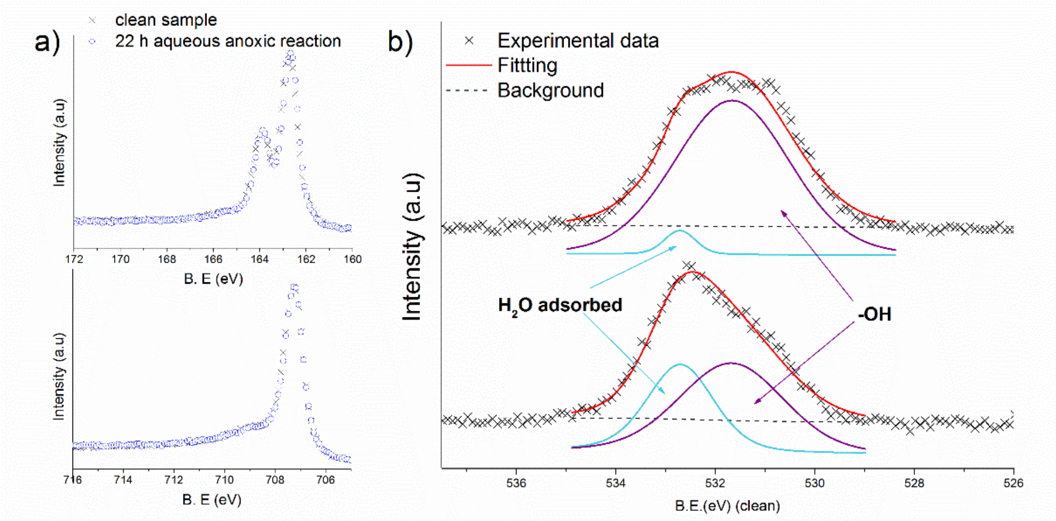

Figure S.5. a) S2p (on the top) and Fe2p3/2 spectra (on the bottom) of clean sample and after 22 hours of aqueous reaction under anoxic conditions; none of the orbitals showed major changes with respect to the clean sample. b) Comparison of O1s spectra for clean sample and after 22 hours of aqueous reaction under anoxic conditions. The increment of the hydroxyl contribution could be associated with the formation of hydrated complexes at iron dangling bonds [3], which could act as precursors for the formation of H_2O_2.

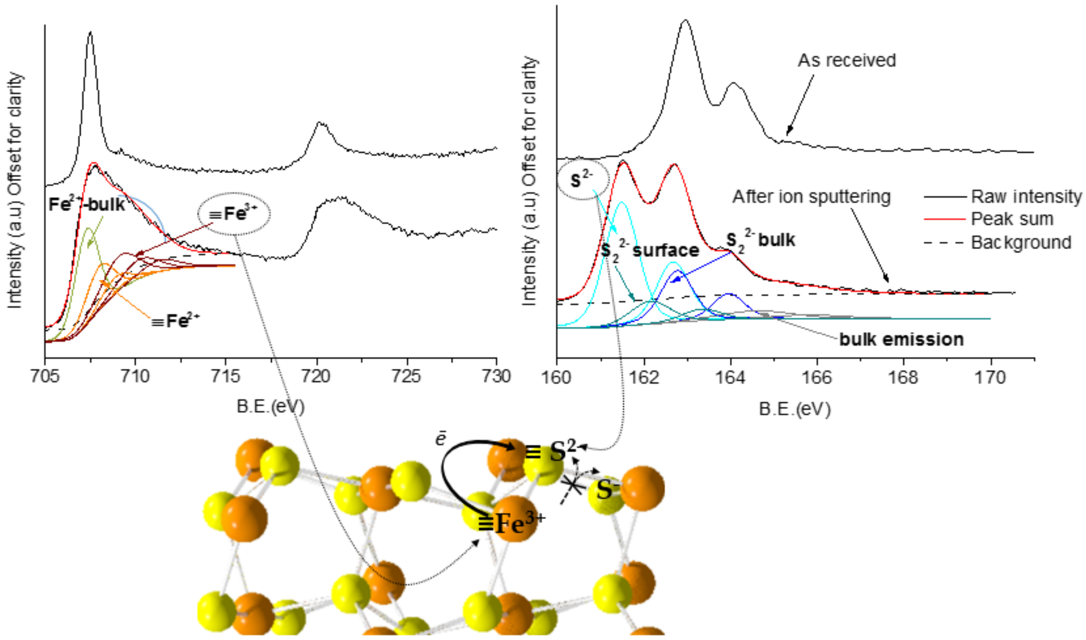

Figure S.6. XPS spectra comparing unreacted (001) pyrite surface before (on the top) and after ion sputtering (on the bottom). In order to facilitate the identification of iron dangling bonds, (001) face of pyrite was ion-sputtered, promoting the breakage of the S-S dimers (as occurs after mechanical grinding). From the rupture of S-S bonds arose new surface species according to the auto-redox reaction $\equiv S^{-} + \equiv Fe^{2+} \rightarrow \equiv S^{2-} + \equiv Fe^{3+}$, resulting in a binding energy shift as is reflected in the XPS spectra. The $Fe2p_{3/2}$ orbital (on the left) showed an increase of the tail to higher binding energy that it was ascribed to iron surface species ($\equiv Fe^{3+}$) whereas in the S2p orbital (on the right) appears a great contribution below 162 eV, that can be assigned to sulfur monomers ($\equiv S^{2-}$)

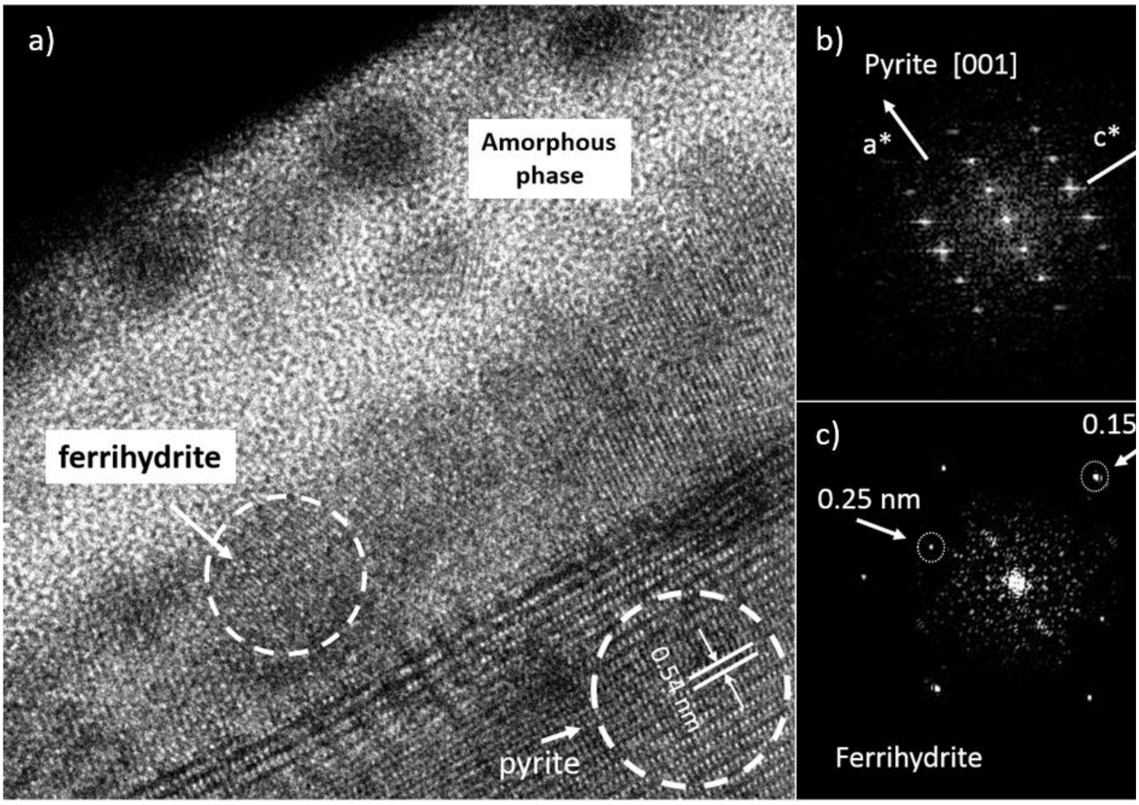

Figure S.7. a) HRTEM image showing the formation of secondary products over a pyrite lamella after 22 hours immersed in a micromolar solution of H_2O_2, under anoxic conditions b) FFT of the crystalline part, showing the diffraction spots arrangement characteristic of pyrite c) FFT of the clusters, showing the interplanar spacing associated with a two-line ferrihydrite [4,5].

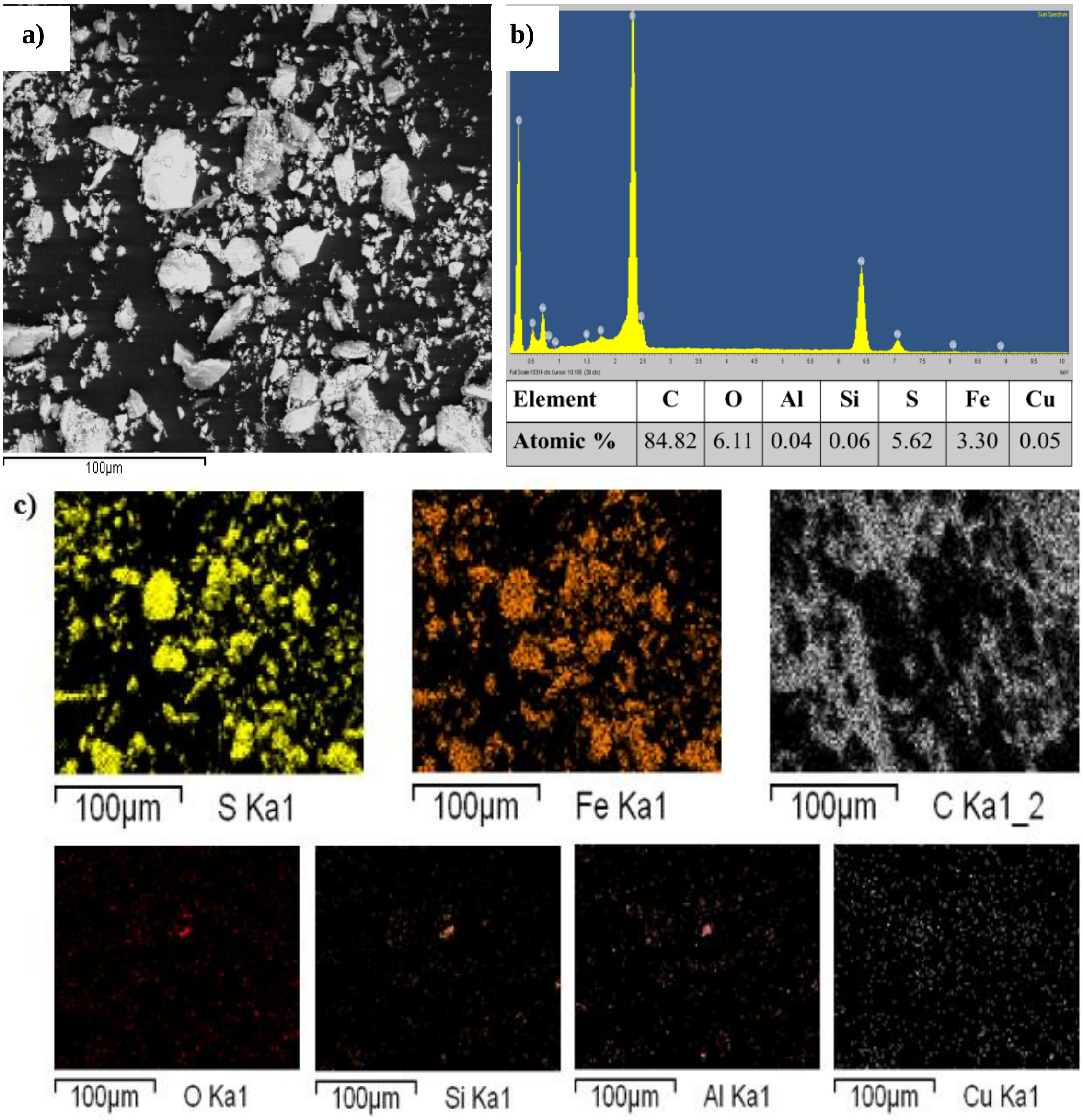

Element	C	O	Al	Si	S	Fe	Cu
Atomic %	84.82	6.11	0.04	0.06	5.62	3.30	0.05

Figure S.8. a) SEM image of pyrite microparticles showing that they are non-uniformly distributed and exhibit irregular shapes terminated by sharp edges. Some ultrafine particles remaine attached in their surface despite of the cleaning treatment. b) EDS complete spectrum collected for the X-Ray Map showing some contribution of Al, Si, O and Cu. C and part of the O emissions are associated with the epoxy resin used to prepare mounted samples. The estimated stoichiometric ratio of S: Fe of about 1.7 indicate S-deficient samples, suggesting the presence of S-vacancies induced by the grinding process [6,7]. c) X-ray Map showing the element distribution of pyrite microparticles. The space distribution of Al, Si, and O suggest that they are related with some small impurities of silicate grains in the samples, which have been shown to be poor producers of Reactive Oxygen Species (ROS) in comparison with pyrite grains [8].

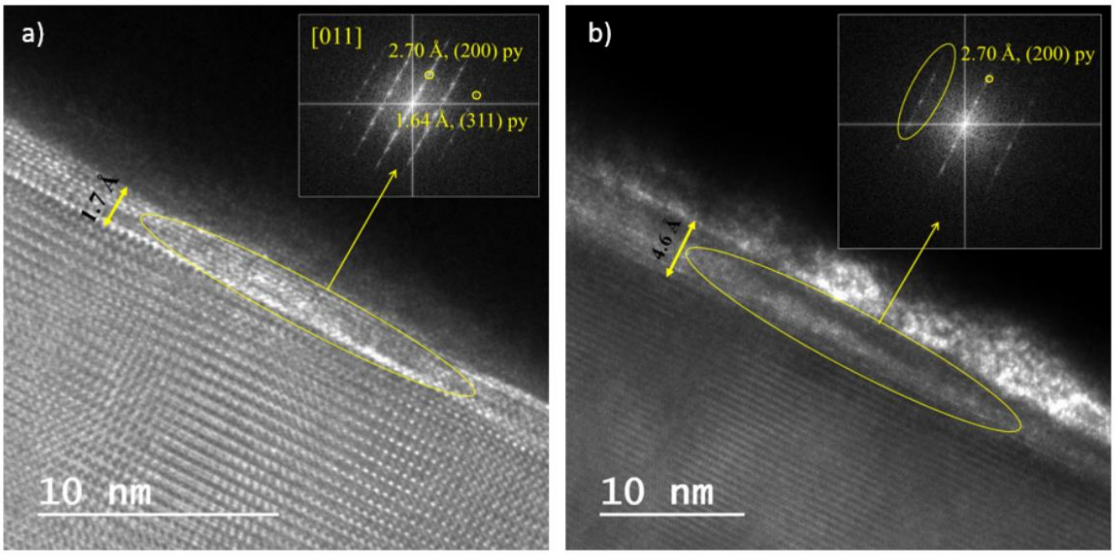

Figure S.9. HRTEM images showing the evolution of pyrite interface after reaction in a micromolar solution of H_2O_2 under anoxic conditions: a) unreacted sample b) reacted sample (22 hours). The alteration layer superimposed with the fresh pyrite structure showed an increment of their thickness (from 1.7 to 4.6Å) together with an increase of their structural disorder.

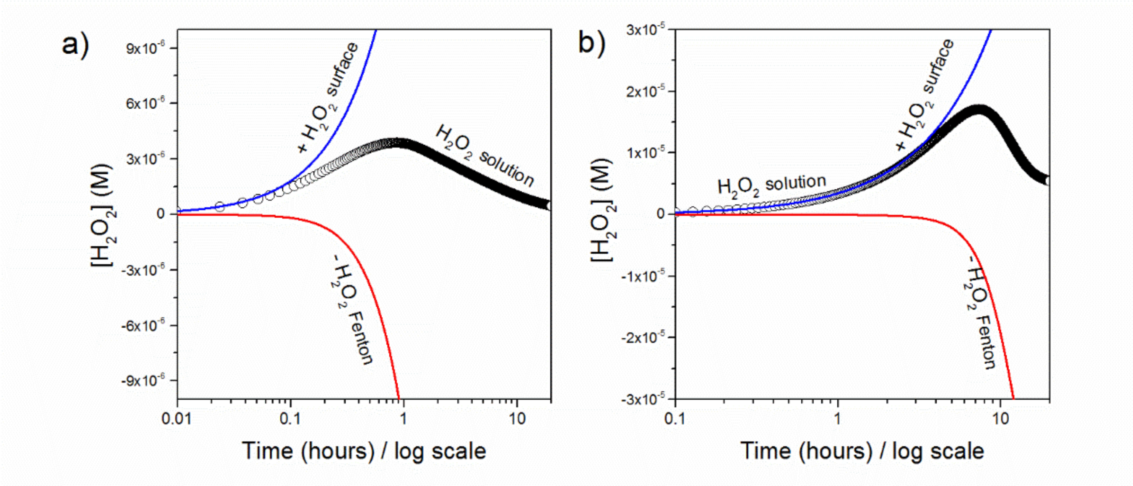

Figure S.10. Deconvolution of H_2O_2 curves in the generation and degradation processes by using the reaction fluxes estimated from the kinetics model, under: a) oxic; and b) anoxic conditions. The maximum amount of H_2O_2 was shifted to longer times in anoxic conditions where the reaction proceeded slowly.

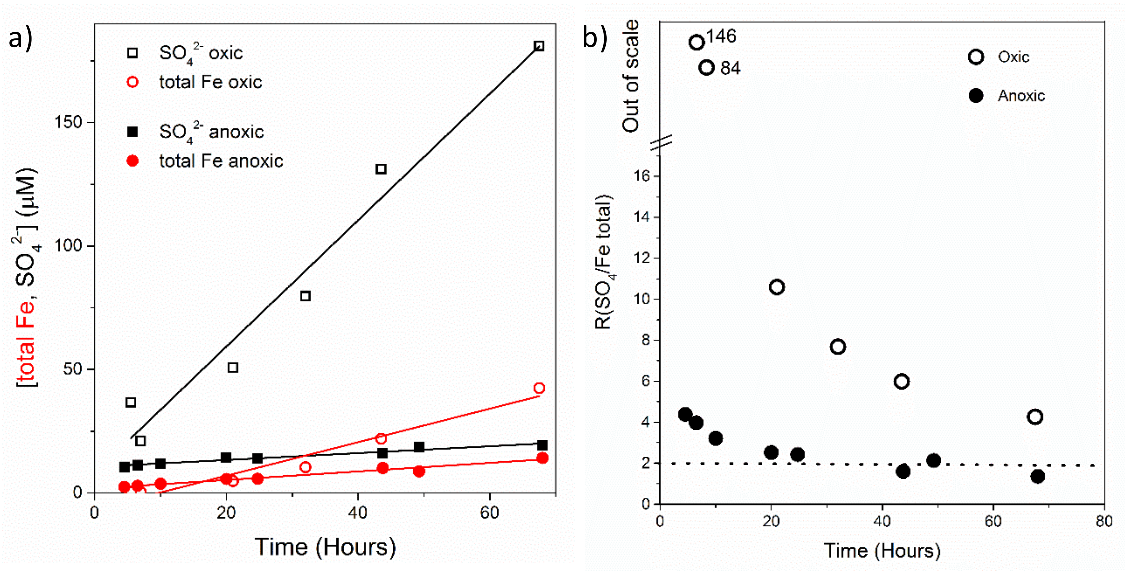

Figure S.11. Total iron and sulfate release. a) Total iron (measured with an Inductively Coupled Plasma, ICP) and sulfate (measured with ion chromatography) released by two pyrite slurries in the dark, under oxic (particle load ~ 0.20 g/L, ΔpH = 6.02-3.44) and anoxic conditions (particle load ~ 0.14 g/L, room temperature, ΔpH = 6.02 – 4.41); b) estimated production rates under oxic and anoxic conditions (assuming a zero-order kinetics: *k*Fetotal = 1.9 x 10^{-10} vs 4.8 x10^{-11}, kSO_4^{2-} = 8.0 x 10^{-10} vs 3.9 x 10^{-11} in $M{\cdot}s^{-1}$, respectively). The observed rates show that the accumulation of both species is one order of magnitude lower in absence of O_2. However, it becomes apparent that without adding an aqueous oxidant, such as dissolved O_2 or Fe^{3+}, and, without illumination, pyrite dissolution still occurs upon contact with H_2O. High concentrations of sulfate were detected at the beginning of each experiment, which has been observed also in previous studies [9]. That may be related to residual sulfate adsorbed to the upper layer of pyrite, formed in the sample cleaning process, which would be easily transferred to the solution.

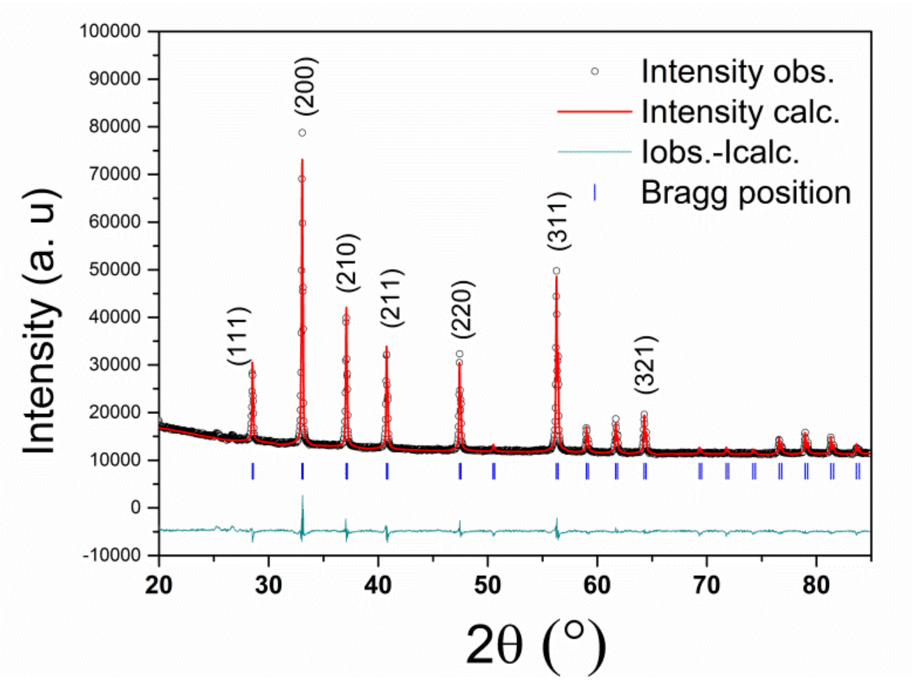

Figure S.12. Rietveld plot of XRD performed on pyrite particles. Experimental data (open circles), fitted data (red line) and difference between them (grey line). Powder diffraction pattern was recorded using CuKα radiation in a Phillips diffractometer equipped with a linear detector and graphite monochromator. An angular range of 2–90º 2θ, with a scan rate of 0.02º min^{-1} was employed for data acquisition. Microstructural parameters (average 'crystallite size' and microstrain) were calculated from the Scherrer method [10] using the Fullproff code [11,12]. Results of this analysis give an average value of 82 nm, for the crystallite size and a micro-strain of $\varepsilon < 0.25$ ‰.

a)

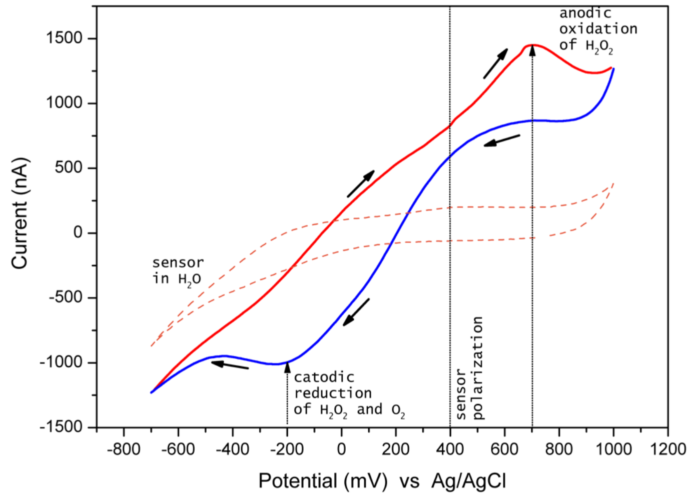

b)

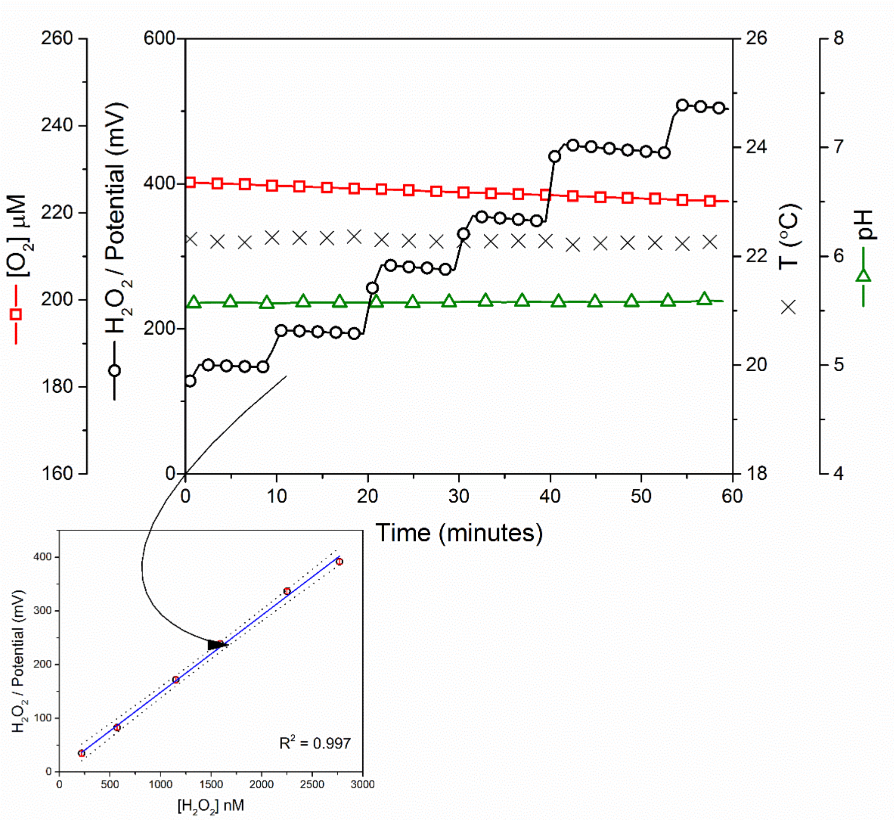

Figure S.13. a) Cyclic voltammetry of an H_2O_2 microsensor in the potential window of -0.8 ~ 1 V with 0.1 V/s of scan rate in neutral water and in 1 mmol H_2O_2 (pH 7); b) example of H_2O_2 calibration in unbuffered neutral water.

a)

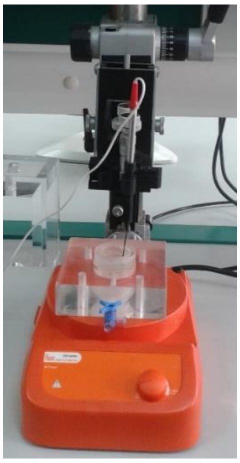

b)

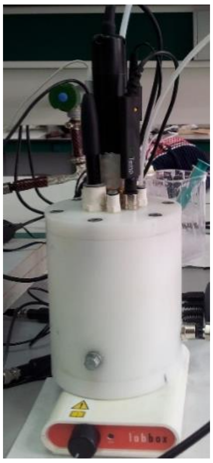

c)

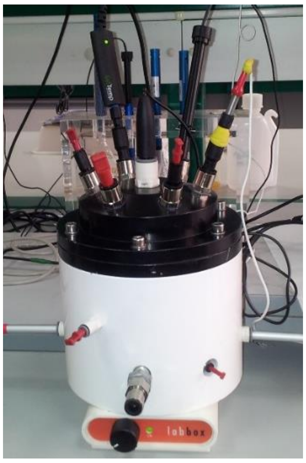

d)

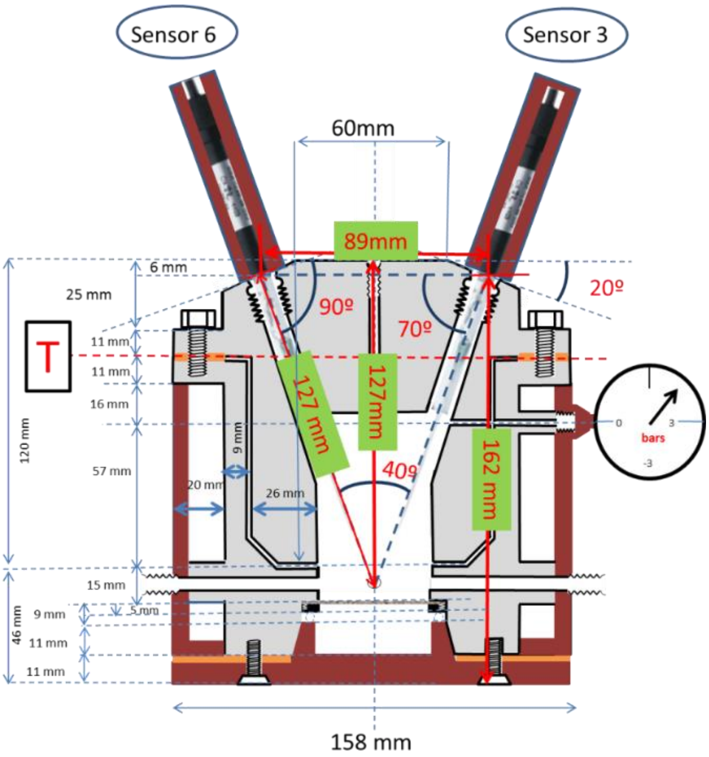

Figure S.14. Batch reactors designed: a) methacrylate chambers to perform oxic-open to the atmosphere experiments under room light condition; b) polyamide and c) aluminum batch reactors to perform oxic - close to the atmosphere and anoxic experiments in dark conditions. d) Schematic view of the aluminum bath reactor. The reactor was coated with an anodized layer to prevent both chemical and electrical interferences. The reactor is equipped with 6 channels on the top to introduce the microsensors that connect directly with the inner chamber where pyrite suspension is injected. The inner chamber also presents two fiber optic connections for simultaneously monitoring UV-VIS spectroscopic data.

a)

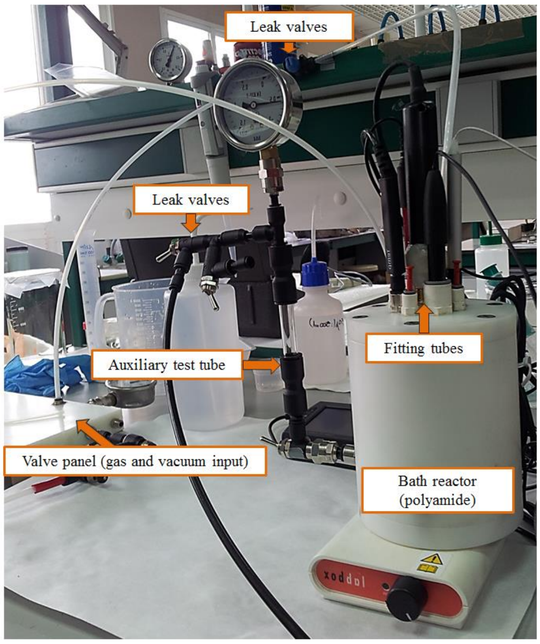

b)

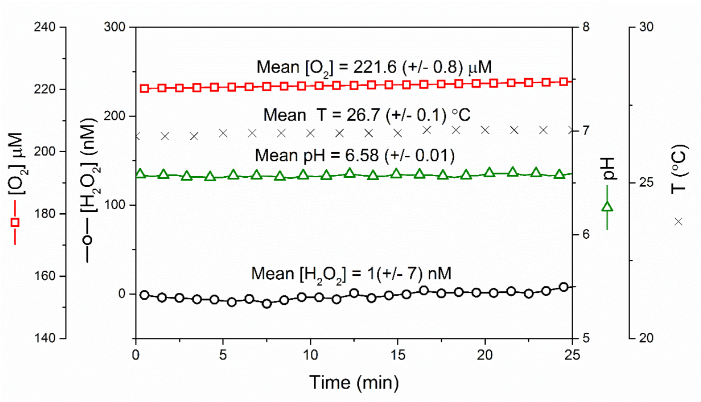

c)

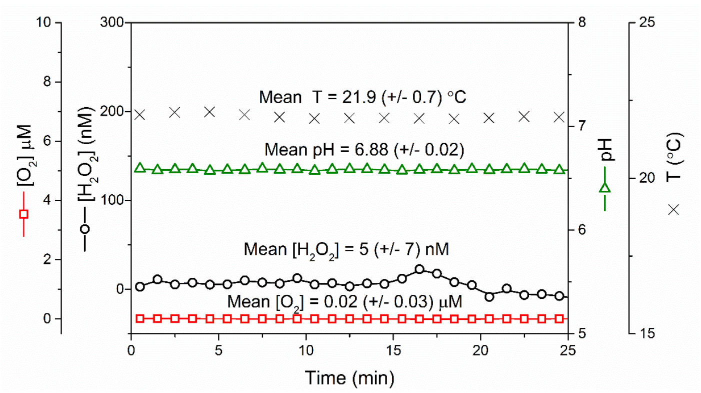

Figure S.15. Experimental set-up. a) Circuit designed for anoxic experiments; hydraulic fitting tubes of different diameters were used to suit each of the sensors to the batch reactor, whereas the flow of water and gases were adjusted by using leak valves and flow regulators, keeping the system isolated. To remove O_2, H_2O was previously purged with N_2 (g) (at least 1h). Pyrite microparticles were stored in an auxiliary test tube connected to the reactor under low vacuum conditions to avoid O_2 adsorption. Once the sensor responses were stable, pyrite particles were injected into the reactor by pressure differences; b) and c) examples of baselines registered before the injection of pyrite under oxic and anoxic conditions, respectively.

a)

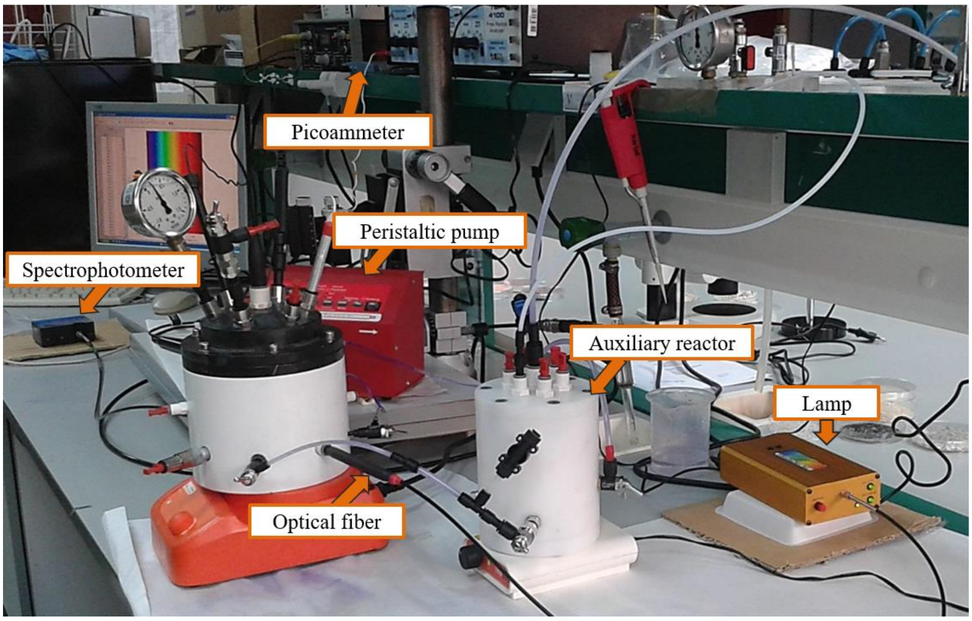

b)

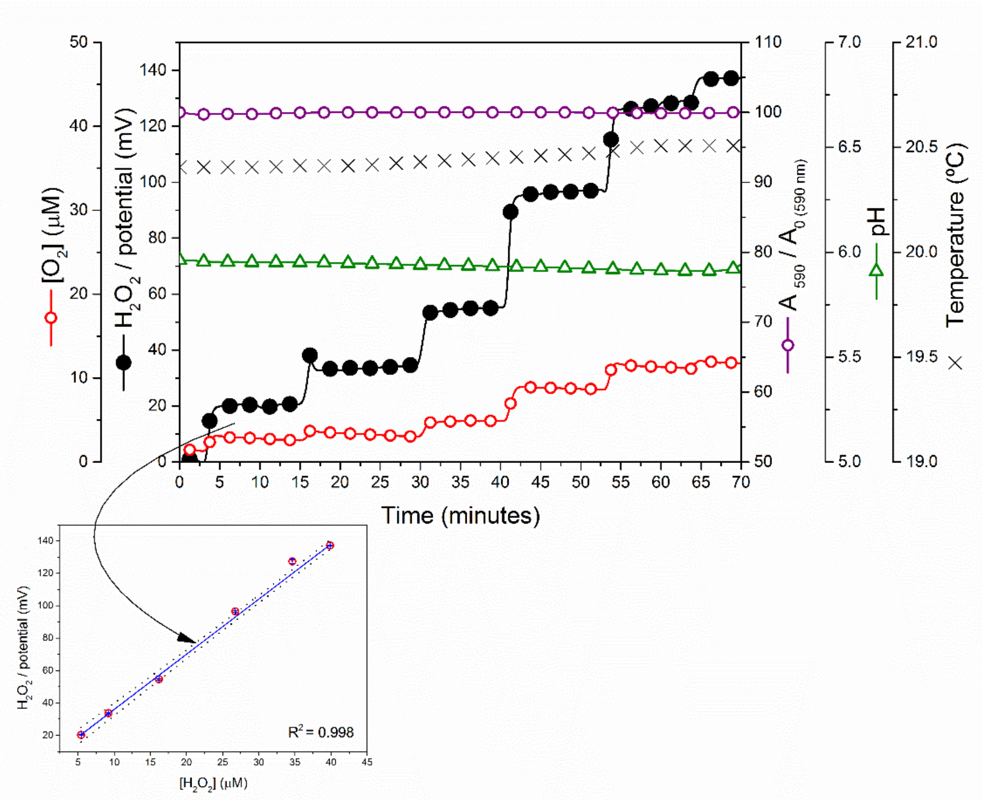

Figure S.16. a) Spectroscopic experiment for OH• detection; b) example of CV stability against several injections of H_2O_2 in micromolar concentration under anoxic conditions.

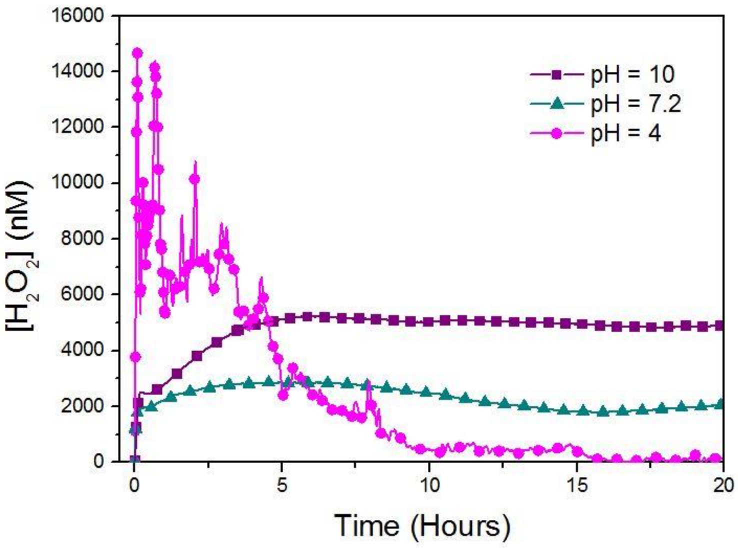

Figure S.17: H_2O_2 curves from pyrite slurries in buffer solutions under oxic conditions (pH =4, load particle = 2.95 g/L; pH = 7.2, load particle = 0.93 g/L; pH = 10, load particle = 1.18 g/L).

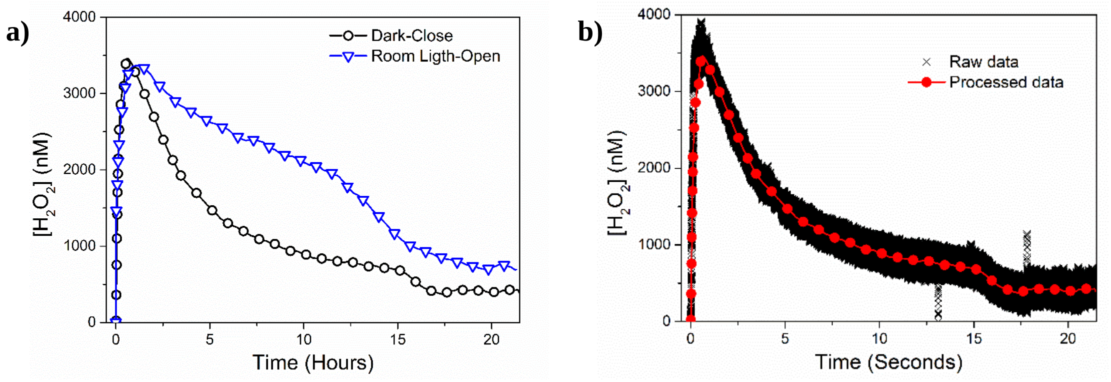

Figure S.18. a) Comparison of H_2O_2 evolution under oxic conditions: open black circles correspond to oxic-close under dark conditions (particle loading = 0.70 g/L) and, open blue triangles to oxic-open under light room conditions (particle loading = 1.00 g/L). The H_2O_2 evolution registered from pyrite slurries in oxic-open under room lab light and oxic-closed under dark conditions falls in the experimental variability registered, suggesting that neither atmospheric pressure of O_2 nor room light exposition are limiting factors for H_2O_2 formation.

b**)** Comparison of raw and processed data for one curve of H_2O_2. Experimental data were reduced and smoothed by moving average (from 1data/second to 1 data/min); plot was divided by sections in order to make more uniform the density of points represnted for aesthetic reasons.

2. Tables.

Table S.1. Reaction scheme of H_2O_2 decomposition employed in the kinetic model.

Reaction	Constants	References
$Fe^{2+} + H_2O_2 \rightarrow Fe^{3+} + OH^{\bullet} + OH^{-}$	$k_1 = 63\ M^{-1}\ s^{-1}$	Fan *et al.* 2009
$Fe^{3+} + H_2O_2 \rightarrow Fe^{2+} + HO_2^{\bullet} + H^{+}$	$k_2 = 0.01\ M^{-1}\ s^{-1}$	
$H_2O_2 + OH^{\bullet} \rightarrow HO_2^{\bullet} + H_2O$	$k_3 = 2.7 \times 10^{7}\ M^{-1}\ s^{-1}$	
$Fe^{3+} + HO_2^{\bullet}/O_2^{\bullet -} \rightarrow Fe^{2+} + H^{+} + O_2$	$k_4 = 2000\ M^{-1}\ s^{-1}$ (3.1 x 10^{7}	
$Fe^{2+} + O_2 \rightarrow Fe^{3+} + O_2^{\bullet -}$	$k = 8 \times 10^{13}\ M^{-2}\ atm^{-1}\ min^{-1}$<br>(pH dependence = $[OH^{-}]^{2}$)	Chandra and Gerson, 2010
$HO_2^{\bullet} = O_2^{\bullet -} + H^{+}$	$K_1 = 3.55 \times 10^{-5}$ M	Sychev and Isak, 1995
$Fe^{3+} + H_2O = Fe(OH)^{2+} + H^{+}$	$K_2 = 2 \times 10^{-3}$ M	

Table S.2. Binding energies assigned to sulfur species in the S2p orbital.

Species	B. E (eV)	References	This study (+/- 0.05 eV)
**S^{2-}**	161.2<br>161.3	Nesbitt *et al.* 2000<br>Schaufuβ *et al.* 1998	161.5
**S_2^{2-} surface**	162.0	Nesbitt *et al.* 2000<br>Schaufuβ *et al.* 1998	162.1
**S_2^{2-} bulk**	162.7	Nesbitt *et al.* 2000;<br>Schaufuβ *et al.* 1998a;<br>Smart *et al.* 1999	162.8
**S_n^{2-}**	161.9-163.2<br>163.8<br>163.0-163.4	Smart *et al.* 1999<br>Schaufuβ *et al.* 1998<br>Buckley and Woods, 1987	163.2
**Energy loss**	164.0	Bronold *et al.* 1994	164.6
**SO_4^{2-}**	168.3<br>168.7	Schaufuβ *et al.* 1998<br>Demoisson *et al.* 2008	168.8

Table S.3. Binding energies assigned to iron species in the Fe $2p_{3/2}$ orbital

Species	B. E (eV)	References	This study (eV)
**Fe^{2+}-bulk**	707.4	Buckley and Woods, 1987	707.3
**Fe^{2+}-surface[a]**	707.1<br>707.6<br>708.2	Nesbitt *et al.* 1998<br>Nesbitt *et al.* 2000<br>Schaufuβ *et al.* 1998	708.0
**Fe^{3+}-surface[a]**	708.9<br>709.1	Nesbitt *et al.* 2000<br>Schaufuβ *et al.* 1998	709.1

[a] Main peak values of the iron multiplets.

3. Modeling approaches.

3.1 Surface generation of H_2O_2

In the presence of O_2, we assume that H_2O_2 formation is mainly triggered by a heterogeneous surface reaction produced by the adsorption of O_2 (g) on surface Fe^{2+}- sites. For the sake of simplicity, we considered the sum of both reactions to describe the H_2O_2 generation at Fe^{2+}-sites:

$$2\,(\equiv \mathrm{PyFe}^{2+}) + \mathrm{O}_{2_{ads}} + 2\mathrm{H}^{+} \rightarrow 2(\equiv \mathrm{PyFe}^{3+}) + \mathrm{H_2O_2} \qquad (1)$$

Several studies have identified the Fe^{2+} oxidation by O_2 on the pyrite surface as the rate-determining step of pyrite dissolution in oxic conditions, but there are still some discrepancies about the adsorption process [13-16]. We assume that O_2 undergoes dissociative adsorption at Fe^{2+}-sites which can be characterized by a Langmuir-Freundlich isotherm [16]:

$$\theta = \frac{K_{ads}[O_2]^{0.5}}{1 + K_{ads}[O_2]^{0.5}} \qquad (2)$$

where, θ and K_{ads} are the coverage degree and the adsorption constant, respectively; a value of K_{ads} = 1.36 m^3/mol was used in the calculations [15]; and the power exponent of 0.5 takes into account the dissociative mechanism during the adsorption process at the monolayer where H_2O_2 is formed.

Finally, we associate the formation of H_2O_2 with the density of Fe^{2+}-sites, according to the following expression:

$$S_{PyFe^{2+}}(t) = \frac{A_o}{V} \cdot \left(\frac{\mathrm{Pyrite}_t}{\mathrm{Pyrite}_0}\right)^n \cdot Fc_{oxic} \qquad (3)$$

where, A_0/V is the initial surface area of pyrite per volume of water (m^2/L); $\left(\frac{\mathrm{Pyrite}_t}{\mathrm{Pyrite}_0}\right)^n$ is a factor for accounting changes of surface area during pyrite dissolution, where Pyrite_0 is the initial moles of pyrite and Pyrite_t is the moles at a given time (calculated for the model according to the pyrite dissolution equation, below), with n = 2/3 assuming cubic particles[17] and; Fc_{oxic}, is a correction factor for limiting the percentage of surface associated with the density of Fe^{2+}-sites. We contemplated a value of 0.25 in the calculations [18-20]. Therefore, the generation of H_2O_2 at Fe^{2+}-sites was defined by:

$$\frac{d[H_2O_2]_{oxic}}{dt} = k_{oxic} \cdot \theta \cdot S_{PyFe^{2+}}(t) \qquad (4)$$

where k_{oxic} is the specific rate constant (mol/m^{2}s) used as the adjustable parameter in the model.

Under anoxic conditions, we assume that the formation of H_2O_2 is triggered by water splitting at pyrite Fe^{3+}-sites, promoting the formation of adsorbed $OH^{\bullet}$ radical that ultimately forms H_2O_2, according to:

$$\equiv PyFe^{3+} + H_2O \rightarrow \equiv\equiv PyFe^{2+} + (OH^{\bullet})_{ads} + H^{+} \quad (5)$$

$$(OH^{\bullet})_{ads} + (OH^{\bullet})_{ads} \rightarrow (H_2O_2)_{aq} \quad (k = 3.6 \times 10^{9}\ M\ s^{-1})\ ^{21}\ (6)$$

We also consider that the formation of H_2O_2 is dependent on the density of Fe^{3+}-sites as in the oxic case, assuming that only 1/3 of the total defect sites (Fc_{anoxic}= 0.083) result from the breaking of S-S bonds [20].

$$S_{PyFe^{3+}}(t) = \frac{A_o}{V} \cdot \left(\frac{Pyrite_t}{Pyrite_0}\right)^{n} \cdot Fc_{anoxic} \quad (7)$$

Therefore, the generation of H_2O_2 at Fe^{3+}-sites was defined by:

$$\frac{d[H_2O_2]_{anoxic}}{dt} = k_{anoxic} \cdot S_{PyFe^{3+}}(t) \quad (8)$$

where k_{anoxic} is the specific rate constant of H_2O_2 formation, in mol/m^{2}s, used as the adjustable parameter in the model.

5.2 Pyrite dissolution

We modeled the production of Fe^{2+} and SO_4^{2-} using the overall stoichiometry equation for pyrite dissolution:

$$FeS_2 + 8H_2O \rightarrow Fe^{2+} + 2SO_4^{2-} + 16H^{+} \quad (9)$$

In oxic conditions, under the pH range of our experiments (from 7 to 3.5), the main oxidant that leads to pyrite dissolution is dissolved O_2 and the kinetic rate law can be estimated according to the following general expression [9, 22]:

$$\frac{dFeS_2}{dt} = k_{pyr} \cdot [O_2]^{0.5} \cdot [H^{+}]^{b} \cdot \frac{A_o}{V} \cdot \left(\frac{Pyrite_t}{Pyrite_0}\right)^{0.67} \quad (10)$$

with, $dFeS_2/dt$ expressed in volume units (mol/L·s); k_{pyr} the specific rate constant = $10^{-7.86}$ in mol/m^2·s; $\left(\frac{A_o}{V}\right)$ is the initial surface area of pyrite per volume of H_2O (m^2/L); $[O_2]$ and $[H^+]$ are the concentrations of dissolved O_2 and H^+ in mol/L and the exponents indicate the reaction order of each species: 0.5 for O_2, and b (-0.21 to 0.14) for H^+.

In anoxic conditions pyrite dissolution should proceed by H_2O_2 oxidation, initially produced at defect sites. The rate of pyrite dissolution by H_2O_2 has been considered to be

linearly dependent on the $[H_2O_2]$ [23,24]. We applied the rate expression given by McKibben and Barnes [24], considering the specific rate constant (k_{pyr}) as an adjustable parameter and including a linear dependence on $[H_2O_2]$:

$$\frac{dFeS_2}{dt} = k_{pyr} \cdot [H_2O_2] \cdot \frac{A_o}{V} \cdot \left(\frac{Pyrite_t}{Pyrite_0}\right)^{0.67} \qquad (11)$$

with $dFeS_2/dt$ expressed in volume units (mol/L·s); k_{pyr} is the specific rate constant in L/m^2·s; $\left(\frac{A_o}{V}\right)$ is the initial surface area of pyrite per volume of H_2O (m^2/L); $[H_2O_2]^n$ in molar units with (n=1).

5.3 Degradation of H_2O_2

We modeled the H_2O_2 decomposition by the free radical sequence initiated by the Fenton reaction, using the rate constants taken from literature (Table S.1). In addition, an initial amount of $[Fe^{2+}]_0$ was included in the model to take into account the heterogeneous Fenton reaction of H_2O_2 with the Fe^{2+} on iron-terminated surface sites. A maximum value of $[Fe^{2+}]_0$ was estimated to be in the order of 10^{-5} (mol/L) on the basis of ≈ 6.78 Fe^{2+} atoms/nm^2 for [001] faces of pyrite (≈ 6.78 x1018 (m^2/L) / 6,023 x 10^{23} =1.12 x 10^{-5} (mol/L)). Despite the fact that we observed discrete Fe^{3+}-patches on the pyrite surface, we assumed that their “passivation” effect is negligible in the aqueous microparticles experiments due to the fast decrease of pH and the stirring conditions.

4. Material and Methods.

4.1 Microsensors

Transient evolution of H_2O_2 was monitored with microsensors (ISO-HPO-100, World Precision Instruments, Inc.). The H_2O_2 microsensors contain a flexible, activated carbon-fiber sensing electrode coated with a proprietary membrane that enhances the low detection limit (LDL) of H_2O_2 to a value of 10 nM (response time < 5 seconds). Applied potential was set at 0.4 V respect to an Ag/AgCl reference electrode. At this polarization voltage, H_2O_2 is detected by the anodic oxidation produced in the working electrode of the microsensor. The signal was amplified with a picoammeter (a four-channel TBR4100 Free Radical Analyzer, World Precision Instruments). Since the potential detected by the redox reaction in the sensor follows a linear relationship with the amount of H_2O_2 in solution, the concentration of this molecule is easily estimated by a linear regression (Figure S.13).

A Clark type microsensor (Unisense DK) was employed to monitor the dissolved $O_{2\,(aq)}$. The advantages of this sensor are the small diameter of its tip, providing a high response time (<0.3 sec), and the low detection limit (LDL of 0.3 µM). This sensor responds linearly to the O_2 concentration and only need two-point calibration (at 0% atm and 100% atm of O_2 saturation) to estimate the concentration of dissolved O_2 in solution. Measurements were taken using a polarization voltage of -0.8 V versus an Ag/AgCl reference electrode. Signal amplification was performed by a picoammeter (Unisense PA3000).

Physicochemical variables (i.e., pH and temperature) were monitored during the time course of the experiments by using a glass pH-meter (Vernier FPH-BTA) and a Pt100 temperature sensor (Vernier).

4.2 Spectroscopic experiments

Spectroscopic experiments were designed to measure Fe^{3+} and $OH^{\bullet}$ species in real time by monitoring absorption bands at specific wavelengths, as follows:

- Fe^{3+} species and Fe^{3+}-oxhydroxides nanoparticles were measured tracking the absorbance bands in the wavenumber range of 300-700 nm. As the amount of Fe^{3+}-complexes formed in these conditions was expected to be low, we used a liquid waveguide capillary flow cell (LWCC; path length: 250 cm; WPI) to increase the spectra resolution.

- The $OH^{\bullet}$ production was measured by the degradation of the crystal violet (CV) as a dye probe that has been shown to be a useful tool to monitor the time-evolution of $OH^{\bullet}$ in several Fenton systems [25-27]. The color of the dye is removed due to the specific oxidation by $OH^{\bullet}$, tracking the decrease of the absorbance band at λ = 590 nm. Experiments were conducted using the bath

reactor itself as the cuvette (path length: 40 mm) (Fig. S.8a). Despite $OH^{\bullet}$ is the main responsible for CV degradation, interaction reactions with other ROS (H_2O_2, $O_2^{\bullet-}$) cannot be totally discarded and thus, we relate the kinetics of CV degradation to the $OH^{\bullet}$ generation from a qualitatively point of view. However, in presence of H_2O_2 at micromolar concentrations (as registered in our experiments) the CV spectra remains nearly constant (Fig. S.16b).

4.3 Cyclic Voltammetry (CV)

In order to highlight the redox reactions taking place through adsorbed H_2O on the surface of pyrite under anoxic conditions we used cyclic voltammetry. Composite electrodes were built on the surface of screen printed Pt-electrodes by immobilizing pyrite nanoparticles (pyrite Np's) coated with a Nafion® membrane [28] to obtain /Pt/Pyrite Np's/Nafion®/ as the working electrode. A graphite was used as the counter electrode, and an Ag/AgCl electrode (3M, KCl) as the reference electrode. Cyclic voltammetry was done using a potentiostat (BVT technologies) over a potential range from -1000 mV to +1400mV V (vs. Ag/AgCl 3M KCl), scan rates of 10 mV s^{-1}. Experiments were made in PBS buffer (pH=7.2) solutions purged with N_2.

Pyrite nanoparticles synthesis has been described elsewhere [29,30]. Prior to electrode preparation, pyrite Np's were acidic washed (0.1M HCl). Manipulations were made under N_2 atmosphere. Isopronanol Nafion suspensions were mixed with pyrite NP's and deposited over the surface electrode. Then, the electrode was dried in vacuum and left for 30 min at 100º C.

In our opinion, this procedure is superior for the determination of redox products taking place at the pyrite interface for various reasons: (1) pyrite Np's offers higher reactive surface compared to single crystal pyrite electrodes. (2) The use of Nafion ® generates an adequate backbone that provides a rapid hydration at the interface, due to the side chains terminated on sulfonic acid, useful to study the redox behavior of adsorbed aqueous species. In addition, its low oxygen permeability limits the passage of $O_2(g)$ as well as negatively charged ions through the membrane. For this reason, if cathodic oxygen reduction (ORR) or anodic oxygen oxidation (OOR) are detected, they should be considered as by-products of primary reactions involving adsorbed species like $H_2O/OH^{\bullet}/H_2O_2$ (being an additional clue about the H_2O_2 formation under anoxic conditions). (3) Using composite bi-electrodes (i.e., Pt-Pyrite Np´s) can help to identify simultaneously redox products derived from pyrite formula together with redox products from free radical species (resulting from adsorbed H_2O), as the electrochemical potentials of the latter has been measured in bare Pt-electrodes and are available in the literature.

4.1 X-Ray photoelectron spectroscopy (XPS).

XPS selective regions were analyzed as follows:

Orbital S2p: Sulfur spectra were analyzed using two spin-orbit, $S2p_{3/2}$ and $S2p_{1/2}$, that produce a doublet with a separation of ~1.19 eV and an intensity ratio of 2:1 [31]. The binding energies of sulfur species used are summarized in Table S.2.

Orbital $Fe2p_{3/2}$: A well-defined peak (707.3 eV) was present in this region, which is associated with the bulk contribution of octahedral-coordinated Fe^{2+}. In addition to this peak, several authors [1,7,20,32] have shown the existence of surface iron species in the neighboring of broken bonds that contain unpaired electrons, resulting in multiplet splitting of their associated photopeaks: (i) the rupture of S-Fe bound gives a Fe^{2+}- multiplet, while (ii) the Fe^{3+}- multiplet are due to the broken bonds of S-S. It is assumed that peaks involved in multiplets contain 3-4 peaks separated by ~ 1 eV with the same FWHM values and their intensities decrease as the binding energies increase. In addition, iron oxides and iron salts also present multiplet patterns [33,34]. Usually, high resolution energy (i.e., synchrotron-XPS) is needed to accurately identify the iron species present in this orbital. Therefore, we analyzed these spectra from a qualitative point of view and only deconvoluted the ion-sputtering sample, where the Fe surface species contribution (Table S.3) increased due to Ar^+ bombardment.

Orbital O1s: Oxygen spectra provide additional information to test the surface oxidation. Assignation of the oxygen species in orbital O1s is complex due to their contributions appear in a narrow range of binding energy. Deconvolution of O1s spectra was done by using only three single contributions at 530.2, 531.7 eV and 532.7 eV associated with oxides, hydroxyl or sulfoxy species and adsorbed water, respectively [35] (NIST database values, http://srdata.nist.gov/xps/default.aspx).

References

1 Bronold, M., Tomm, Y. & Jaegermann, W. Surface states on cubic d-band semiconductor pyrite (FeS2). *Surface Science* **314**, L931-L936, doi:10.1016/0039-6028(94)90230-5 (1994).

2 Kendelewicz, T., Doyle, C. S., Bostick, B. C. & Brown, G. E. Initial oxidation of fractured surfaces of FeS2 (100) by molecular oxygen, water vapor, and air. *Surface Science* **558**, 9, doi:10.1016/j.susc.2004.03.045 (2004).

3 Weerasooriya, R., Makehelwala, M. & Bandara, A. Probing reactivity sites on pyrite-oxidative interactions with 4-chlorophenol. *Colloids and Surfaces A: Physicochemical and Engineering Aspects* **367**, 65-69, doi:10.1016/j.colsurfa.2010.06.023 (2010).

4 Banfield, J. F., Welch, S. A., Zhang, H., Ebert, T. T. & Penn, R. L. Aggregation-Based Crystal Growth and Microstructure Development in Natural Iron Oxyhydroxide Biomineralization Products. *Science* **289**, 751-754, doi:10.1126/science.289.5480.751 (2000).

5 Drits, V. A., Sakharov, B. A., Salyn, A. L. & Manceau, A. Structural model for ferrihydrite. *Clay Minerals* **28**, 185-207, doi:10.1180/claymin.1993.028.2.02 (1993).

6 Krishnamoorthy, A., F.W. Herbert, S. Yip, K.J. Van Vliet, B. Yildiz, Herbert, F. W., Sidney, Y., Krystyn, J. V. V. & Bilge, Y. Electronic states of intrinsic surface and bulk vacancies in FeS2. *Journal of Physics: Condensed Matter* **25**, 045004, doi:10.1088/0953-8984/25/4/045004 (2012).

7 Nesbitt, H. W., Bancroft, G. M., Pratt, A. R. & Scaini, M. J. Sulfur and iron surface states on fractured pyrite surfaces. *American Mineralogist* **83**, 1067-1076, doi:10.2138/am-1998-9-1015 (1998).

8 Jones, G. C., Becker, M., van Hille, R. P. & Harrison, S. T. L. The effect of sulfide concentrate mineralogy and texture on Reactive Oxygen Species (ROS) generation. *Applied Geochemistry*, doi:10.1016/j.apgeochem.2012.11.015 (2012).

9 Liu, R., Wolfe, A., Dzombak, D., Stewart, B. & Capo, R. Comparison of dissolution under oxic acid drainage conditions for eight sedimentary and hydrothermal pyrite samples. *Environ Geol* **56**, 171-182, doi:10.1007/s00254-007-1149-0 (2008).

10 Scherrer, P. in *Kolloidchemie Ein Lehrbuch* 387-409 (Springer Berlin Heidelberg, 1912).

11 Rodríguez-Carvajal, J. FULLPROF: A Program for Rietveld Refinement and Pattern Matching Analysis. *Abstracts of the Satellite Meeting on Powder Diffraction of the XV Congress of the IUCr, Toulouse, France*, 127, doi:citeulike-article-id:1840305 (1990).

12 Roisnel, T. & Rodríquez-Carvajal, J. WinPLOTR: A Windows Tool for Powder Diffraction Pattern Analysis. *Materials Science Forum* **378-381**, 118-123, doi:10.4028/www.scientific.net/MSF.378-381.118 (2001).

13 Chandra, A. P. & Gerson, A. R. The mechanisms of pyrite oxidation and leaching: A fundamental perspective. *Surface Science Reports* **65**, 293-315, doi:10.1016/j.surfrep.2010.08.003 (2010).

14 Lowson, R. T. Aqueous oxidation of pyrite by molecular oxygen. *Chemical Reviews* **82**, 461-497, doi:10.1021/cr00051a001 (1982).

15 Nicholson, R. V., Gillham, R. W. & Reardon, E. J. Pyrite oxidation in carbonate-buffered solution: 1. Experimental kinetics. *Geochimica et Cosmochimica Acta* **52**, 1077-1085, doi:10.1016/0016-7037(88)90262-1 (1988).

16 Jeppu, G. P. & Clement, T. P. A modified Langmuir-Freundlich isotherm model for simulating pH-dependent adsorption effects. *Journal of Contaminant Hydrology* **129–130**, 46-53, doi:10.1016/j.jconhyd.2011.12.001 (2012).

17 Parkhurst, D. L. & Appelo, C. A. J. *User's guide to PHREEQC (version 2) [microform] : a computer program for speciation, batch-reaction, one-dimensional transport, and*

inverse geochemical calculations / by David L. Parkhurst and C.A.J. Appelo. (U.S. Geological Survey : Earth Science Information Center, 1999).

18 Guevremont, J. M., Strongin, D. R. & Schoonen, M. A. A. Effects of surface imperfections on the binding of CH3OH and H2O on FeS2(100): using adsorbed Xe as a probe of mineral surface structure. *Surface Science* **391**, 109-124, doi:10.1016/s0039-6028(97)00461-5 (1997).

19 Murphy, R. & Strongin, D. R. Surface reactivity of pyrite and related sulfides. *Surface Science Reports* **64**, 1-45, doi:DOI: 10.1016/j.surfrep.2008.09.002 (2009).

20 Nesbitt, H. W. *et al.* Synchrotron XPS evidence for Fe2+-S and Fe3+-S surface species on pyrite fracture-surfaces, and their 3D electronic states. *American Mineralogist* **85**, 850-857, doi:10.2138/am-2000-5-628 (2000).

21 Pastina, B. & LaVerne, J. A. Effect of Molecular Hydrogen on Hydrogen Peroxide in Water Radiolysis. *The Journal of Physical Chemistry A* **105**, 9316-9322, doi:10.1021/jp012245j (2001).

22 Williamson, M. A. & Rimstidt, J. D. The kinetics and electrochemical rate-determining step of aqueous pyrite oxidation. *Geochimica et Cosmochimica Acta* **58**, 5443-5454, doi:10.1016/0016-7037(94)90241-0 (1994).

23 Antonijević, M. M., Janković, Z. D. & Dimitrijević, M. D. Kinetics of chalcopyrite dissolution by hydrogen peroxide in sulphuric acid. *Hydrometallurgy* **71**, 329-334, doi:10.1016/S0304-386X(03)00082-3 (2004).

24 McKibben, M. A. & Barnes, H. L. Oxidation of pyrite in low temperature acidic solutions: Rate laws and surface textures. *Geochimica et Cosmochimica Acta* **50**, 1509-1520, doi:10.1016/0016-7037(86)90325-X (1986).

25 Alshamsi, F. A., Albadwawi, A. S., Alnuaimi, M. M., Rauf, M. A. & Ashraf, S. S. Comparative efficiencies of the degradation of Crystal Violet using UV/hydrogen peroxide and Fenton's reagent. *Dyes and Pigments* **74**, 283-287, doi:10.1016/j.dyepig.2006.02.016 (2007).

26 Fan, H.-J. *et al.* Degradation pathways of crystal violet by Fenton and Fenton-like systems: Condition optimization and intermediate separation and identification. *Journal of Hazardous Materials* **171**, 1032-1044, doi:10.1016/j.jhazmat.2009.06.117 (2009).

27 Sirés, I., Guivarch, E., Oturan, N. & Oturan, M. A. Efficient removal of triphenylmethane dyes from aqueous medium by in situ electrogenerated Fenton’s reagent at carbon-felt cathode. *Chemosphere* **72**, 592-600, doi:10.1016/j.chemosphere.2008.03.010 (2008).

28 Giovanni, C. D. *et al.* Low-Cost Nanostructured Iron Sulfide Electrocatalysts for PEM Water Electrolysis. *ACS Catalysis* **6**, 2626-2631, doi:10.1021/acscatal.5b02443 (2016).

29 Li, W. *et al.* Pyrite nanocrystals: shape-controlled synthesis and tunable optical properties via reversible self-assembly. *Journal of Materials Chemistry* **21**, 17946-17952, doi:10.1039/c1jm13336e (2011).

30 Gil-Lozano, C., Losa-Adams, E., Davila, F. A. & Gago-Duport, L. Pyrite nanoparticles as a Fenton-like reagent for in situ remediation of organic pollutants. *Beilstein journal of nanotechnology* **5**, 855-864, doi:10.3762/bjnano.5.97 (2014).

31 Smart, R. S. C., Skinner, W. M. & Gerson, A. R. XPS of sulphide mineral surfaces: metal-deficient, polysulphides, defects and elemental sulphur. *Surface and Interface Analysis* **28**, 101-105, doi:10.1002/(sici)1096-9918(199908)28:1<101::aid-sia627>3.0.co;2-0 (1999).

32 Schaufuß, A. G. *et al.* Reactivity of surface chemical states on fractured pyrite. *Surface Science* **411**, 321-328, doi:10.1016/s0039-6028(98)00355-0 (1998).

33 Gupta, R. P. & Sen, S. K. Calculation of multiplet structure of core p-vacancy levels. *Physical Review B* **10**, 71-77, doi:10.1103/PhysRevB.10.71 (1974).

34 Gupta, R. P. & Sen, S. K. Calculation of multiplet structure of core p -vacancy levels. II. *Physical Review B* **12**, 15-19, doi:10.1103/PhysRevB.12.15 (1975).

35 Pratt, A. R., Muir, I. J. & Nesbitt, H. W. X-ray photoelectron and Auger electron spectroscopic studies of pyrrhotite and mechanism of air oxidation. *Geochimica et Cosmochimica Acta* **58**, 827-841, doi:10.1016/0016-7037(94)90508-8 (1994).
